# The effects of perspective taking primes on the social tuning of explicit and implicit views toward gender and race

**DOI:** 10.3389/fpsyg.2023.1014803

**Published:** 2023-03-02

**Authors:** Jeanine Lee McHugh Skorinko, Craig DiGiovanni, Katherine Rondina, Amy Tavares, Jennifer Spinney, Mariam Kobeissi, Luisa Perez Lacera, Daniel Vega, Paul Beatty, Melissa-Sue John, Aidan Doyle

**Affiliations:** ^1^Psychological and Cognitive Sciences Program, Social Science and Policy Studies Department, Worcester Polytechnic Institute, Worcester, MA, United States; ^2^Penn State Health, Hershey Medical Center, Penn State Harrisburg, Middletown, CT, United States; ^3^Department of Psychology, American University, Washington, DC, WA, United States; ^4^Department of Psychology, Family, and Justice Studies, University of Saint Joseph, West Hartford, CT, United States; ^5^Department of Psychological Sciences, University of Connecticut, Storrs, CT, United States

**Keywords:** perspective taking, social tuning, implicit attitudes, self-stereotyping, explicit attitudes

## Abstract

The current research aims to investigate whether perspective taking influences social tuning, or the alignment of one’s self-views, explicit attitudes, and/or implicit attitudes with those of an interaction partner. In six different experiments, participants believed they would interact with a partner to complete a task. Prior to this ostensible interaction, participants were given a perspective taking mindset prime, or not, and information about their ostensible interaction partners views. Participants then completed attitude measures related to the partner’s perceived views. Experiments 1a, 1b, and 2 examined whether perspective taking with an ostensible interaction partner who endorses gender traditional (or non-traditional) views align their self-views with this partner, including implicit self-views (Experiment 2). Experiments 3–5 investigated whether perspective taking leads to social tuning for egalitarian racial attitudes, including when the partner’s expectations of how others will be and when the participant learns their ostensible IAT score at the beginning of the session. We predicted perspective takers would be more likely to social tune their explicit and implicit attitudes to the attitudes of their interaction partner than non-perspective takers. Across all experiments, perspective takers were more likely to social tune their self-views and explicit attitudes than non-perspective takers. However, social tuning never occurred for implicit attitudes. Thus, future research is needed to understand why perspective taking does not influence the tuning of implicit attitudes, but other motivations, like affiliative and epistemic, do.

## Introduction

Attitudes once thought to be stable and intrapersonal can be malleable to social and cultural contexts (Smith and Semin, 2004; Mesquita et al., 2010). Implicit, or indirectly measured, attitudes can also change, at least in the short-term, though the changes may be weak (Forscher et al., 2019; Greenwald and Lai, 2020). One way in which attitudes may change in social situations is through social tuning, or the unconscious alignment of an individual’s beliefs with those of an interaction partner (Davis and Rusbult, 2001; Sinclair et al., 2005a,b; Lun et al., 2007; Bechtoldt et al., 2010; Huntsinger and Sinclair, 2010; Skorinko et al., 2015; Skorinko and Sinclair, 2018). One factor that is postulated to be relevant in social tuning but has not been empirically explored is perspective taking (Sinclair et al., 2005a). Perspective taking can influence interpersonal interactions (Boca et al., 2018) and implicit and explicit attitudes (Goldstein and Cialdini, 2007; Skorinko et al., 2012; Todd and Galinsky, 2014). However, perspective taking can backfire (Skorinko and Sinclair, 2013) and highlight differences rather than similarities (Hodges et al., 2018). Given the mixed effects perspective taking might have on the transmission of implicit and explicit attitudes, the current research investigates the role perspective taking plays in the social tuning of self-views, explicit, and implicit attitudes.

### Social tuning of implicit and explicit self-views and attitudes

Research shows that social tuning in interpersonal contexts facilitates explicit and implicit belief transmission and maintenance (Higgins and Rholes, 1978; Echterhoff et al., 2005; Sinclair and Lun, 2006; Weisbuch et al., 2009). The social tuning model contends that this alignment happens to achieve a sense of mutual understanding, or shared reality (Hardin and Higgins, 1996; Hardin and Conley, 2001; Sinclair et al., 2005a,b). Moreover, results indicate that it is the interpersonal nature that drives social tuning of implicit and explicit attitudes (Lun et al., 2007; Skorinko and Sinclair, 2018). For instance, social tuning of implicit racial attitudes only occurred when presented with the interaction partner’s beliefs and not to similar messages in the social environment (e.g., a poster; Lun et al., 2007). Similarly, heavier women showed increased implicit self-esteem when their interaction partner endorsed positive body attitudes; but a week later, this only held if they interacted with the same partner (Weisbuch et al., 2009).

Social tuning of implicit and explicit self-views and attitudes occurs when individuals experience affiliative motivation, epistemic motivation, or endorse a collectivistic mindset (Lun et al., 2007; Skorinko et al., 2015; Skorinko and Sinclair, 2018). For instance, females who experienced high affiliative motivation and believed their partner valued gender traditional women rated themselves as more gender traditional than females with low affiliative motivation (Sinclair et al., 2005a). Likewise, white participants who experienced high epistemic motivation showed less implicit prejudice when the experimenter wore a shirt that said “Eracism” than white participants with less epistemic motivation (Lun et al., 2007). Research also shows that individuals with a collectivistic background or mindset held less implicit and explicit prejudice toward sexual orientation when their experimenter wore a shirt endorsing treating others equally than those with an individualistic background or mindset (Skorinko et al., 2015). Self-presentation, from a restrictive perspective (Jones and Pittman, 1982; Sinclair et al., 2005a), is unlikely to explain social tuning as participants always learned their partner would never learn about the participants beliefs.

### Perspective taking, interpersonal relationships, and shared reality

Perspective taking is the ability to imagine the thoughts of another individual and to take into consideration the way the individual views the world (Cooley, 1902; Mead, 1925, 1936; Piaget, 1926; Selman, 1980; Davis, 1983). As such, interpersonally, perspective taking can help individuals anticipate another person’s reactions and behaviors in different situations and may facilitate communication between people (Davis, 1983; Krauss and Fussell, 1996; Krauss and Chiu, 1998; Boca et al., 2018). It can also assist conflict resolution and impact social relationships and satisfaction (Falk and Johnson, 1977; Franzoi et al., 1985; Long, 1993; Bissonnette et al., 1997; Harvey and Omarzu, 1997, 1999; Takaku et al., 2001; Kaplan and Maddux, 2002; Reis et al., 2004). This research indicates that perspective taking in a close interpersonal relationship can lead to alignment between partners and may increase mutual understanding; however, it is unclear what happens in social interactions where the relationship is not yet formed.

Shared Reality theory argues that individuals will try to develop a mutual understanding to form (and also maintain) interpersonal relationships (Hardin and Higgins, 1996; Hardin and Conley, 2001; Sinclair et al., 2005a). This means sharing reality is relevant to interactions with strangers as it is about the formation of a relationship. One way in which individuals may form shared reality is by trying to take their partners perspective to get a better understanding of what their partner knows and to help shape their interaction (Sinclair et al., 2005a). However, Hodges et al. (2018) also point out that perspective taking may impede and inhibit shared reality because it may highlight differences more than similarities. Thus, while perspective taking is postulated to influence social tuning and shared reality, empirical research is needed to understand the role perspective taking plays in the process of social tuning.

### Perspective taking and explicit and implicit intergroup attitudes

While the role perspective taking has in the social tuning has yet to be explored, past work has examined the role perspective taking plays in intergroup attitudes. The results of this work are mixed (see Paluck et al., 2021 for a review on prejudice reduction). Some work finds that perspective takers, compared to non-perspective takers, use fewer stereotypes (Galinsky and Moskowitz, 2000; Scaffidi Abbate et al., 2020), express more positive explicit and implicit attitudes toward stigmatized groups (Vescio et al., 2003; Todd et al., 2011; Shih et al., 2013; Todd and Burgmer, 2013; Gutierrez et al., 2014; Todd and Galinsky, 2014; Weyant, 2019), increase their social coordinator of behavior with the target (Galinsky et al., 2008; Ku et al., 2010), or are more likely to help an outgroup member (Shih et al., 2009). For instance, perspective takers showed less implicit bias toward Black individuals (Todd et al., 2011) and Hispanic individuals (Weyant, 2019) than non-perspective takers.

In contrast, research also demonstrates that perspective taking can increase stereotypic views of outgroup members (Galinsky and Ku, 2004; Vorauer et al., 2009; Paluck, 2010; Skorinko and Sinclair, 2013; Vorauer and Sasaki, 2014). For instance, people reported more negative stereotypes of the elderly if they took the perspective of an elderly man that confirmed stereotypes (e.g., sick) than did not clearly confirm stereotypes (Skorinko and Sinclair, 2013). In addition, individuals who imagine the other during perspective taking reported more prejudice than those who imagined the self (Vorauer and Sasaki, 2014). Moreover, perspective taking may not always lead to reductions in implicit bias (Aberson and Haag, 2007; Todd and Simpson, 2016; Edwards et al., 2017). For example, perspective takers only showed reduced implicit prejudice if the target matched their gender-race prototype (e.g., Black man or Asian woman; Todd and Simpson, 2016).

Much of the past work involves perspective takers learning about a target, that they know they will never meet, through a photograph, an audio recording, or a video (Batson et al., 1997, 2002; Stephan and Finlay, 1999; Finlay and Stephan, 2000; Galinsky and Moskowitz, 2000; Vescio et al., 2003; Dovidio et al., 2004; Galinsky et al., 2005, 2008; Shih et al., 2009, 2013; Laurent and Myers, 2011; Todd et al., 2011; Skorinko and Sinclair, 2013; Gutierrez et al., 2014; Vorauer and Sasaki, 2014; Huang et al., 2021). Thus, it is unclear whether these effects will hold during social interactions.

While a few studies involved an ostensible interaction, none investigated whether perspective takers social tune. Todd et al. (2011) investigated how perspective takers treated a Black female experimenter, but it did not examine whether the partner’s perceived views influenced participant’s attitudes. In addition, participants were led to believe they would be interacting with another participant, not the experimenter. Vorauer and Sasaki (2014) led participants to believe that they would be exchanging written information with a partner who was an outgroup member (i.e., Chinese Canadian or Aboriginal Canadian). The researchers examined whether the type of perspective taking (e.g., imagine-self or imagine-other) led to reductions in prejudice. However, the participants did not learn about the partner’s perceived views, so no social tuning could occur. Across both studies, the partner is always an outgroup member. Therefore, it is unclear whether explicit or implicit attitudes will be influenced when the partner does not belong to the outgroup.

### Perspective taking and self-views

Past work on social tuning has also found alignment of self-views with an interaction partner. Perspective taking also influences self-views. For example, participants who visualized an older family member prior to reading an article on premarital sex reported more conservative self-views toward sex when primed to perspective take than those not primed to perspective take (Skorinko et al., 2012). Perspective taking can also prompt individuals to see more overlap between themselves and another person (i.e., self-other overlap), even if this person is an outgroup member (Davis et al., 1996; Galinsky et al., 2005; Goldstein and Cialdini, 2007; Hodges et al., 2011; Laurent and Myers, 2011; Skorinko et al., 2012). The self-other overlap with a target mediates changes in perspective takers’ self-concepts and endorsement of other’s beliefs (Laurent and Myers, 2011). Based on this past work, it seems likely that perspective taking with the perceived views of an interaction partner should also lead to the social tuning of self-views.

### Current research

Perspective taking can be an important conduit in interpersonal relationships, intergroup attitudes, and self-views. However, the role perspective taking plays in the transmission of implicit and explicit attitudes through interpersonal processes, like social tuning, have yet to be empirically explored. Thus, the current research investigates the role perspective taking plays in the social tuning of explicit and implicit self-views and intergroup attitudes.

To do this, participants in six experiments are led to believe they will interact with an ostensible interaction partner. Participants are then primed to perspective take (or not) using a mindset prime (Skorinko et al., 2012) because past work indicates that activating mindsets can influence implicit associations (Sassenberg and Moskowitz, 2005; Forscher et al., 2019). They also learn about the perceived views of their partner in a subtle manner (e.g., survey responses completed by ostensible partner, the tshirt the partner is wearing). Participants than complete explicit (all experiments) and implicit (Experiments 2–5) measures to assess social tuning toward their partner.

Experiments 1a, 1b, and 2 examine whether female perspective takers social tune when a partner expresses traditional (or non-traditional) beliefs about women by measuring explicit self-feminine ratings (all three experiments) and implicit self-feminine ratings using a subliminal priming task (Experiment 2). Experiment 1b also examines the role of affiliative motivation and perspective taking on social tuning. Experiments 3, 4, and 5 examine whether perspective takers social tune when a partner endorses egalitarian racial views (e.g., wears an “Eracism” t-shirt) by measuring explicit racial attitudes (i.e., items from the Modern Racism Scale and the Symbolic Racism Scale) and implicit racial views using the Race Implicit Association Task (IAT; Experiments 3 and 4) or a subliminal priming measure (Experiment 5). Experiments 4 and 5 also investigate factors that may influence perspective taker’s social tuning. Experiment 4 examines whether social tuning occurs to the perceived views or the perceived expectations the partner has of others. Experiment 5 investigates if knowing one’s own ostensible implicit racial beliefs influences social tuning. Based on past research on social tuning and perspective taking, we predict that perspective takers will be more likely to social tune their self-views, explicit, and implicit attitudes that non-perspective takers.

## Experiment 1a

We set out in Experiment 1a to pilot a conceptual replication of previous social tuning research (Sinclair et al., 2005a) to see if perspective taking leads to social tuning. Since perspective taking can lead to changes in self-views and self-other overlap (Davis et al., 1996; Goldstein and Cialdini, 2007; Galinsky et al., 2008; Laurent and Myers, 2011), Experiment 1a investigates if perspective takers will social tune by self-stereotyping more than non-perspective takers. Conceptually replicating Sinclair et al. (2005a), female participants believed they would be interacting with a partner on a task for 5 min to induce low affiliative motivation. Participants were then primed to perspective take (or not) and led to believe their partner held gender traditional views about women. We predicted that perspective takers would social tune by endorsing more feminine self-views than non-perspective takers.

## Method

### Participants

Forty-four females (32 White; 4 Black, 4 Asian, 2 Latino, 1 Biracial, and 1 Unreported) from a Mid-Atlantic University completed the experiment for course credit. The majority (73%) were first year undergraduate students (21% second year; 2% third year; 4% fourth or fifth year undergraduates). All participants gave informed consent prior to participating.

### Design and materials

This experiment utilized a one-way (Perspective taking: Perspective taking vs. None) between-participants design with feminine trait self-ratings as the key dependent measure. The ostensible partner always endorsed gender traditional views.

#### Ostensible interaction and perceived views

Participants learned they were going to interact with an ostensible partner. Since Experiment 1a explored the role of perspective taking and not affiliative motivation, all participants believed they would interact for 5 min which was used to manipulate low affiliative motivation in the past (Sinclair et al., 2005a). To manipulate the ostensible views of the interaction partner, we used the same packet used in Sinclair et al. (2005a) which contained the ostensible partner’s name (Lauren Anderson), gender (female), and supposed responses to three different scales. All scales were 7-point Likert-Type scales with “1” being “Strongly Disagree” or “Not at All” and “7” being “Strongly Agree” “Very Much.”

The Attitudes toward Women scale included four items from Spence et al. (1973) Attitudes toward Women scale and five items from the Benevolent Sexism Inventory (BSI; Glick and Fiske, 1996). It was completed to clearly indicate traditional attitudes toward women. For example, the partner strongly agreed (i.e., circled a “7”) with gender traditional statements such as, “Women should be cherished and protected by men,” and agreed less (i.e., circled a “3”) with gender non-traditional statements such as, “I like women who are assertive and confident.” To disguise the purpose of the experiment, we also included an Attitudes toward Black individuals scale that had 14-items from the Modern Racism Scale (McConahay, 1986) and a personality scale that contained 16 traits (e.g., arrogant, good, happy, moody, and talkative). These scales were completed to suggest neutral views toward Black individuals and a neutral personality.

#### Perspective taking manipulation

Perspective taking is often evoked by instructing people to imagine a target’s thoughts while viewing a photograph and writing a day in the life essay, listening to an audio clip, or watching a video with the target (Batson et al., 1997, 2002; Stephan and Finlay, 1999; Finlay and Stephan, 2000; Galinsky and Moskowitz, 2000; Vescio et al., 2003; Dovidio et al., 2004; Galinsky et al., 2005, 2008; Shih et al., 2009, 2013; Laurent and Myers, 2011; Todd et al., 2011; Skorinko and Sinclair, 2013; Gutierrez et al., 2014; Vorauer and Sasaki, 2014). However, this seems less likely to occur in a social interaction. Rather, it seems more likely something in the situation will subtly activate or prime the mindset. Sentences unscrambling tasks were developed to subtly activate mindsets (Srull and Wyer, 1979). Mindset priming has also been used in previous social tuning experiments (Sinclair et al., 2005a; Lun et al., 2007; Skorinko et al., 2015). Therefore, we utilize a sentence unscrambling task developed in previous research to prime participants with perspective taking or neutral mindsets (Skorinko et al., 2012). In this task, participants unscramble a series of words to make a sentence; however, one of the words is not used in the sentence. Half of the participants completed the perspective taking version that contained 20 sentences related to perspective taking. For instance, “I world blimp her through eyes see the” unscrambled into “I see the world through her eyes.” The other half of the participants completed a neutral version unrelated to perspective taking (same 20 sentences used in Chartrand and Bargh, 1996). For example, “ball throw toss silently the” unscrambled into, “Toss the ball silently.”

#### Feminine self-ratings/self-stereotyping measure

To measure self-stereotyping, participants completed a questionnaire that included 33 personality traits. We embedded eight pretested feminine traits: caring, compassionate, complaining, emotional, faithful, feminine, moody, and talkative. The scores on these eight traits were averaged together (Cronbach *α* = 0.68). Higher scores indicate more feminine/gender traditional self-views (or more self-stereotyping).

#### Self-other overlap measure

The amount of overlap an individual sees between themselves and another person may be heightened during perspective taking (Davis et al., 1996; Galinsky and Moskowitz, 2000; Goldstein and Cialdini, 2007; Laurent and Myers, 2011). Thus, we measured the self-other overlap the participants felt with their partner. To do so, participants rated their first impression of their ostensible partner on the same traits they rated themselves on (adapted from Galinsky and Moskowitz, 2000).

#### Perceived views measure

To make sure that participants picked up on the perceived views of the ostensible partner, participants indicated on a 7-point Likert-type score “how much their partner valued gender traditional people” and also “how much their partner valued gender non-traditional people” (1 = Not At All; 7 = Very Much).

#### Perspective taking, affiliation and self-presentation measures

At the very end of the study, we also measured participants: likelihood to perspective take in two different ways (see Appendix A for items), level of affiliation with their partner, and self-presentation tendencies. All items utilized a 7-point Likert-type scale (1 = Not at All; 7 = Very Much). We measured the natural propensity to perspective using six items from the Perspective Take subscale from the Interpersonal Reactivity Index (IRI; Davis, 1980). We also measured perspective taking with the partner with five items assessing the motivation, the importance, ability, ease of perspective taking. To measure affiliation with the partner, participants indicated (a) how likeable does your partner seem, (b) how motivated are you to get along with your partner, and (c) to what extent do you feel that you and your partner have things in common (Cronbach *α* = 0.74). Finally, we measured self-presentation by assessing how important it was for participants to feel as though their partner liked them and how much they prepared for interaction by imagining how partner would see them (Cronbach *α* = 0.67).

### Procedure

Female participants came into the lab for a study investigating how rumors spread. The participant learned that another (ostensible) participant had arrived a few minutes earlier in another room, and that after each participant completed some individual tasks, they would then interact for 5 min on a task (low affiliative motivation, Sinclair et al., 2005a). The experimenter explained that they would each complete a packet of information about themselves, and while the participant would get to see their partner’s information, the partner would never see the participant’s information. We explained this was because this is how information spreads in the real world. This was done to limit demand characteristics. Participants always learned that their partner, Lauren Anderson, was another female student who endorsed gender traditional views of women. This packet was pre-completed by the research team and did not reflect a real person.

After reading the packet, participants completed a “pilot” task for another study under the guise that we were trying to ensure enough time passed in between learning information about the partner and the interaction, as we explained was often the case in the real world when hearing a rumor about someone and then interacting with that person. This was our perspective taking manipulation. Participants unscrambled words to create 20 sentences that were related to perspective taking or something neutral.

After the perspective taking prime, participants learned that the experiment was on the transmission of rumors, and we wanted to mimic this situation as best we could so they would complete a “randomly selected” questionnaire that may be similar or different to the one their ostensible partner completed. Participants were reminded that their partner would never see their responses. Participants rated themselves on 33 traits, rated their interaction partner on the same traits, provided demographic information (e.g., gender, ethnicity, year in school), and answered questions regarding their beliefs about their partner and the upcoming interaction to measure perceived view perceptions, perspective taking, affiliation and self-presentation. To limit self-presentation, participants placed their completed questionnaire into an envelope, sealed it, and put it in a locked box. After the questionnaire was in the locked box, the experimenter informed participants that there was no interaction, and the study was done. Participants were thanked, debriefed, and awarded course credit.

## Results and discussion

### Perceived view manipulation check

Participants picked up on the perceived views of their partner as they believed their partner valued gender traditional people (*M* = 5.9, *SD* = 1.0) more than gender nontraditional people (*M* = 3.1, *SD* = 1.0), *t*(43) = 10.57, *p* > 0.001, *d* = 1.8, 95% CI [2.3, 3.4], two-tailed test.

### Feminine ratings

Participants’ feminine self-ratings were submitted to a one-way ANOVA with the perspective taking prime as the independent variable. Perspective taker rated themselves as more feminine (*M* = 4.9; *SD* = 0.4) than non-perspective takers (*M* = 4.5 *SD* = 0.6), *F* (1, 42) = 4.25, *p* = 0.045, *η^2^* = 0.092, 95% CI [0.007, 0.71], two-tailed test. The effect remained statistically significant with a bootstrap analysis with 1,000 samples (*p* = 0.02). See Supplementary Table 1.

### Measures of self-other overlap, perspective taking, affiliation, and self-presentation

To examine self-other overlap, we conducted a repeated measures ANOVA with the self and partner feminine ratings as the within variables and the perspective taking condition as the between-participants variable. Contrary to past research where perspective taking increased self-other overlap (Galinsky and Moskowitz, 2000; Goldstein and Cialdini, 2007; Laurent and Myers, 2011), the results showed no statistically significant difference in feminine traits ascribed to oneself compared to the partner (*p* = 0.419), or an interaction between feminine ratings and perspective taking (*p* = 0.587). See Supplementary Table 2.

To examine perspective taking, affiliation, and self-presentation, we conducted one-way ANOVAs for each dependent measure. Looking at the IRI subscale, there were no differences in the propensity to perspective take, *p* = 0.663. There were also no differences in perspective taking with partner, *p* = 0.325. There was no statistically significant effect for affiliation with partner, *p* = 0.074. There was also no statistically significant effect of perspective taking on self-presentation, *p* = 0.703 (*p* = 0.632). See Supplementary Table 3.

### Experiment 1a conclusion

In sum, perspective takers were more likely to social tune, and self-stereotype by endorsing more feminine self-views, than non-perspective takers. Perspective taking did not increase affiliation with the partner or self-presentation.

## Experiment 1b

Experiment 1a provides preliminary evidence that perspective taking leads to social tuning. Since perspective taking is postulated as being a potential mechanism in fulfilling affiliative needs (Hardin and Conley, 2001; Sinclair et al., 2005a) and since perspective taking did not influence affiliation in Experiment 1a, we wanted to more directly investigate the role that affiliative motivation and perspective taking play in social tuning. Therefore, affiliative motivation is added as an independent variable so its influence can be more thoroughly investigated. Based on past research, we predict that those with high affiliative motivation should social tune (Skorinko and Sinclair, 2018) more than those with low affiliative motivation. Based on Experiment 1a results, we also predict that perspective takers, regardless of their affiliative motivation, will social tune more than non-perspective takers.

## Method

### Participants

Seventy-one females (47 White, 10 Asian, 4 Black, 4 Latinx, 4 Multiracial, and 2 Unreported) at a medium-sized private institution in the northeast participated. Participants were evenly split across their year in school (24% first year; 21% second year; 24% third year; 27% fourth or fifth year; 4% unreported) All participants gave informed consent prior to participating and received course credit.

### Design and materials

This experiment utilized a 2 (Perspective taking: Perspective taking, None) x 2 (Affiliative Motivation: Low, High) between-participants design with self-feminine ratings as the key dependent measure. The ostensible partner always endorsed gender traditional views.

#### Perspective taking condition

Instead of the sentence unscrambling task used in Experiment 1a, we adapted the mindset priming procedure used in previous social tuning work (Sinclair et al., 2005a). Participants read and responded to two prompts. Half the participants received prompts related to perspective taking where they had to: (1) put themselves in the shoes of a friend having a rough day, and (2) imagine how they would portray what it was like to be their friend in a movie they were creating. Half the participants received prompts unrelated to perspective taking where they had to imagine: (1) they were at the zoo and described what did, and (2) that they forgot their grocery store list and explained what they would do to recreate the list. We pretested eight different prompts to determine how likely they were to induce perspective taking or affiliation. The two perspective taking prompts were chosen because they induced perspective taking but not affiliation. The neutral prompts used did not induce perspective taking or affiliation.

#### Affiliative motivation condition

Affiliative motivation was manipulated by how long participants believed they would interact with the ostensible partner because past work has found that longer (i.e., 30 min) interactions prompt more affiliative motivation than shorter interactions (i.e., 5 min; Sinclair et al., 2005a). Adapting from this past work, half the participants learned that they would be working with a partner for 5 min to create a 500-word persuasive essay (low affiliative motivation), and half the participants learned they would be working with a partner for 30 min to create a 1,200-word persuasive essay (high affiliative motivation).

#### Feminine traits and other measures

We used the same 8-item feminine ratings as in Experiment 1a (Cronbach’s *α* = 0.64). As in Experiment 1a, participants also rated their first impression of their ostensible partner on the same traits to measure self-other overlap. We used the same items as in Experiment 1a to measure perceived views, perspective taking with the partner (Cronbach *α* = 0.87), affiliation (Cronbach *α* = 0.82), and self-presentation (Cronbach *α* = 0.50). Since the natural propensity to perspective take did not influence the results in Experiment 1a, we did not measure the natural propensity to perspective take through the IRI subscale.

### Procedure

After providing informed consent, participants learned that study investigated individual versus partner writing. Participants believed they would first complete a writing task by themselves, and then would be paired with another (ostensible) participant to complete a persuasive writing task together. Participants learned that they would work with their partner for either 30 min to write a 1,200-word essay (high affiliative motivation) or for 5 min and write a 500-word essay (low affiliative motivation). Participants then selected a slip of paper out of a bowl without looking to supposedly determine the topic they would write about. All slips had the gender traditional topic: “A wife’s primary duties should be caring for her children and household.” The experimenter told the participant that since they got to choose the topic, their partner would select which side of the topic (for or against) they would write, and then they took the topic to the ostensible partner.

While the partner was selecting the side of the topic, participants completed their individual writing task, which was the perspective taking manipulation. Participants read two prompts and wrote a response to each prompt. Half the participants were prompted to put themselves in the shoes of a friend (perspective taking), and the other half were prompted to describe generic tasks they would engage in (no perspective taking). Upon completion of this task, the researcher gave participants the topic sheet that was supposedly completed by the partner. The partner denoted that they wanted to write in favor of the gender traditional statement; hence; signaling that they endorsed gender-traditional views.

Participants then completed the same questionnaire as in Experiment 1a that measured their feminine self-ratings, their partner ratings, their affiliation and perspective taking with partner, self-presentation, demographic information (e.g., ethnicity/race, year in school), and any suspicions. Upon completing the questionnaire, participants learned that there would be no interaction, and they were thanked, debriefed, and awarded credit.

## Results and discussion

### Perceived view manipulation check

Participants picked up on the gender traditional perceived views of the partner, as they believed their partner valued gender traditional people (*M* = 6.2, *SD* = 0.9) more than gender nontraditional people (*M* = 2.5, *SD* = 1.2), *t*(69) = 17.72, *p* > 0.001, *d* = 1.7, 95% CI [3.3, 4.1], two-tailed test.

### Main analyses

We conducted a 2 (Perspective taking: Perspective Taking or No Perspective Taking) x 2 (Affiliative motivation: 5 min, 30 min) between-participants ANOVA on the dependent measures.

#### Feminine ratings

Contrary to our prediction, there was no statistically significant main effect for Affiliative Motivation (*p* = 0.800). There was also no interaction between Affiliative Motivation and Perspective Taking (*p* = 0.565). However, as predicted, there was a statistically significant main effect for perspective taking, such that perspective takers (*M* = 5.2; *SD* = 0.7) endorsed more gender traditional self-views than non-perspective takers (*M* = 4.7; *SD* = 0.6), *F* (1, 67) = 9.02, *p* = 0.004, *η^2^* = 0.119, 95% CI [0.15, 0.76], two-tailed test. The main effect for perspective taking held when bootstrapped with 1,000 samples (*p* = 0.01). See Supplementary Table 4.

While the interaction was not statistically significant, we wanted to examine the effect of perspective taking with and without affiliation. Therefore, we conducted an exploratory analysis to examine the effects of perspective taking (with and without affiliative motivation) and the control condition. To do so, we created a new independent variable that included the following conditions: No Perspective Taking and No Affiliative Motivation (Control), Perspective Taking and No Affiliative Motivation, and Perspective Taking and Affiliative Motivation. The one-way ANOVA was statistically significant, *F* (2, 51) = 3.25, *p* = 0.047, *η^2^* = 0.113, two-tailed test. Pairwise comparisons revealed that perspective takers without affiliative motivation (*M* = 5.3, *SD* = 0.7) rated themselves as more feminine than non-perspective takers (*M* = 4.7; *SD* = 0.7), *t*(51) = 2.48, *p* = 0.016, 95% CI [0.10, 0.99], two-tailed test. Perspective Takers with affiliative motivation (*M* = 5.1, *SD* = 0.7) marginally social tuned more than non-perspective takers (*M* = 4.7; *SD* = 0.7), *t*(51) = 1.78, *p* = 0.081, 95% CI [−0.05, 0.89], two-tailed test. There was no statistically significant difference between the two perspective taking conditions, *p* = 0.573. These effects remained the same after bootstrapping for 1,000 samples.

### Self-other overlap, perspective taking, affiliation, and self-presentation

We conducted a repeated measures ANOVA with the self and partner traits ratings as the within variables and the perspective taking and affiliative motivation conditions as the between participants variables to examine self-other overlap. There was a statistically significant effect for the feminine ratings, *F* (1, 66) = 86.50, *p* < 0.001, *η^2^* = 0.567, 95% CI [0.844, 1.31], two-tailed test (see Supplementary Table 5). Participants rated themselves as more feminine (*M* = 5.0; *SD* = 0.7) than their partner (*M* = 3.9; *SD* = 0.7). There were no interactions between the self-other overlap and perspective taking or affiliative motivation. We conducted 2-way ANOVAs for the remaining measures. As in Experiment 1a, there were no statistically significant effects for perspective taking with partner, felt affiliation, or self-presentation. See Supplementary Table 6.

### Experiment 1b conclusion

As predicted perspective takers engaged in social tuning and self-stereotyped more than non-perspective takers. Contrary to our predictions and past work, affiliative motivation did not lead to social tuning in Experiment 1b. Though those in the non-perspective taking and low affiliative motivation rated themselves as the least feminine. In sum, Experiment 1b provides additional evidence that perspective taking leads to social tuning, but it does not appear to influence affiliation.

## Experiment 2

While Experiments 1a and 1b provide evidence that perspective taking leads to social tuning, neither experiment investigated social tuning of implicit views. In Experiment 2, we address this by adding a subliminal priming task to measure implicit self-views. We also changed the perceived views of the interaction partner to be more gender non-traditional to examine if social tuning still occurs. Based on the results of Experiments 1a and 1b, we predict that perspective takers will endorse less feminine traits than non-perspective takers when they believe their partner supports gender non-traditional beliefs by wearing a Rosie the Riveter shirt.

## Method

### Participants

Sixty-six females (37 White, 7 Black, 13 Asian, 4 Latinx, 1 Middle Eastern and 4 Multi-racial) from a mid-Atlantic university completed the experiment for course credit. The majority (70%) were in their first year of college (23% were second years; 4% were third years; and 3% were fourth or fifth year undergraduates). Four participants were removed for being an outlying response or for being suspicious about the experiment. The analyses are based on 62 female participants (35 White, 6 Black, 13, Asian, 3 Latinx, 1 Middle Eastern, and 4 Multi-Racial). All participants gave informed consent prior to participating.

### Design and materials

This experiment utilized a 2 (Perspective Taking Prime: Perspective Taking vs. No Perspective Taking) × 2 (Perceived Views: Gender Nontraditional vs. Neutral Views) between-participants design with explicit and implicit feminine ratings as measures.

#### Perceived views

The interaction partner is the experimenter. To subtly manipulate the perceived views of the experimenter, they were randomly assigned to wear one of two t-shirts during the experimental session: either a Rosie the Riveter shirt with the slogan “We Can Do It” (i.e., gender nontraditional views) or a plain shirt with no images or slogans (e.g., no gendered view). Pretesting of the Rosie the Riveter shirt indicated that the shirt signals that the person wearing the shirt holds less feminine, more feminist, and more gender nontraditional views. To make sure participants processed the messaging on the shirt, participants completed an eye test where they read the words on the shirt (or a random string of letters for plain shirt condition) as the experimenter feigned that the eyechart went missing (adapted from Lun et al., 2007). Using the t-shirt that the experimenter wears as a perceived views manipulation has been used in past work (Lun et al., 2007; Weisbuch et al., 2009; Skorinko et al., 2015).

#### Perspective taking manipulation

We used the same sentence unscrambling task used in Experiment 1a.

#### Subliminal priming measure

To measure implicit self-views, we use a subliminal priming measure. In this task, participants kept their eyes fixated on a dot in the middle of the screen. They had to indicate as quickly as possible whether the stimulus that appeared was a word (by pressing the “F” key that was relabeled with a “W” sticker) or a nonword (by pressing the “J” key that was relabeled with a “N” sticker). Prior to a stimulus appearing in the middle of the screen, a subliminal prime was presented. Participants were subliminally primed with words related to the self (i.e., “I,” “self,” “me”) or unrelated to the self (i.e., “that,” “at,” “a”). Each prime was forward masked with a nonsensical array of letters (i.e., “jksivlpqmzb”) for 300 ms, the prime exposure lasted 15 ms, and the backward mask (the array of letters) lasted for 300 ms (as in Skorinko et al., 2012).

Participants completed a practice round with a blank prime to become familiar with the task. During the subliminally primed trials, participants were primed with either a “self” word or a “non-self” word. They then indicated whether the stimulus that appeared was a word or nonwork as quickly as possible. Participants completed 60 trials and saw each word and non-word two times, once with the “self” prime and once with the “non-self” prime.

There were 5 positive feminine words (i.e., caring compassionate, faithful, attractive, sensitive), 5 negative feminine words (i.e., complaining, dependent, moody, shy, weak), 5 masculine positive words (i.e., athletic, confident, assertive, powerful, strong), 5 masculine negative words (i.e., aggressive, arrogant, insensitive, selfish, stubborn), and 10 nonwords (e.g., bouie, cirtive, piproe, jojii, vrem). The latency for each stimulus presented was first log transformed. Then the logged latencies for each category of stimulus were averaged together to create a composite score for each category of stimuli (e.g., “self” prime feminine positive, “non-self” prime feminine positive, etc.).

#### Feminine traits and other measures

We used the same eight feminine traits as in Experiments 1a and 1b (Cronbach *α* = 0.54). We also used the same items for perceived views, perspective taking (Cronbach *α* = 0.82), affiliation (Cronbach *α* = 0.66), and self-presentation measure (Cronbach *α* = 0.50). Since self-other overlap did not work in the previous experiments, we did not measure it in Experiment 2. Participants also reported demographic information (e.g., ethnicity/race, year in school, etc.).

### Procedure

When a female participant arrived at the lab, they were greeted by a female experimenter who was wearing either a Rosie the Riveter or a plain shirt. After giving informed consent, participants learned that the experiment investigated the relationship between cognitive skills and personality. Participants were led to believe that they needed to get their eyes checked prior to completing a computer task. As the experimenter looked for the eye chart in a cabinet, they feigned that it was missing and asked if they could improvise by having the participants read the words on the shirt (Rosie shirt condition) or a random string of letters they created on a piece of paper (plain shirt condition). All participants agreed to the eye-test and read the slogan or letters. The eye test was conducted to ensure participants saw the message on the shirt (adapted from Lun et al., 2007).

After the eye test, participants completed a cognitive skills task that was the sentence unscrambling task used in Experiment 1a to manipulate perspective taking. Participants then completed the subliminal priming measure on the computer. After finishing that task, they learned that the remaining personality measure was confidential and that after completing it they should put it in the envelope provided, seal it, and place it in the locked box. This questionnaire was the same one used in Experiments 1a and 1b and included the feminine trait measure, demographics, perceived views, perspective taking, affiliation, and self-presentation measures. After putting the questionnaire in the locked box, participants were thanked, debriefed, and awarded credit.

## Results and discussion

### Perceived views manipulation check

We conducted a repeated measures ANOVA with the participant’s beliefs about how much their partner valued gender traditional and non-traditional people as within factors and the perceived views as the between factor. There was no difference in perceptions of traditionality (*p* = 0.323). There was no interaction between gender traditional and non-traditional ratings with the perceived views (*p* = 0.979). See Supplementary Table 7.

### Main analyses

We conducted a 2 (Perspective Taking Prime: Perspective Taking vs. Neutral) x 2 (Perceived Views: Rosie t-shirt vs. Plain t-shirt) between-participants ANOVA on dependent measures.

### Feminine trait ratings

**Figure 1:**
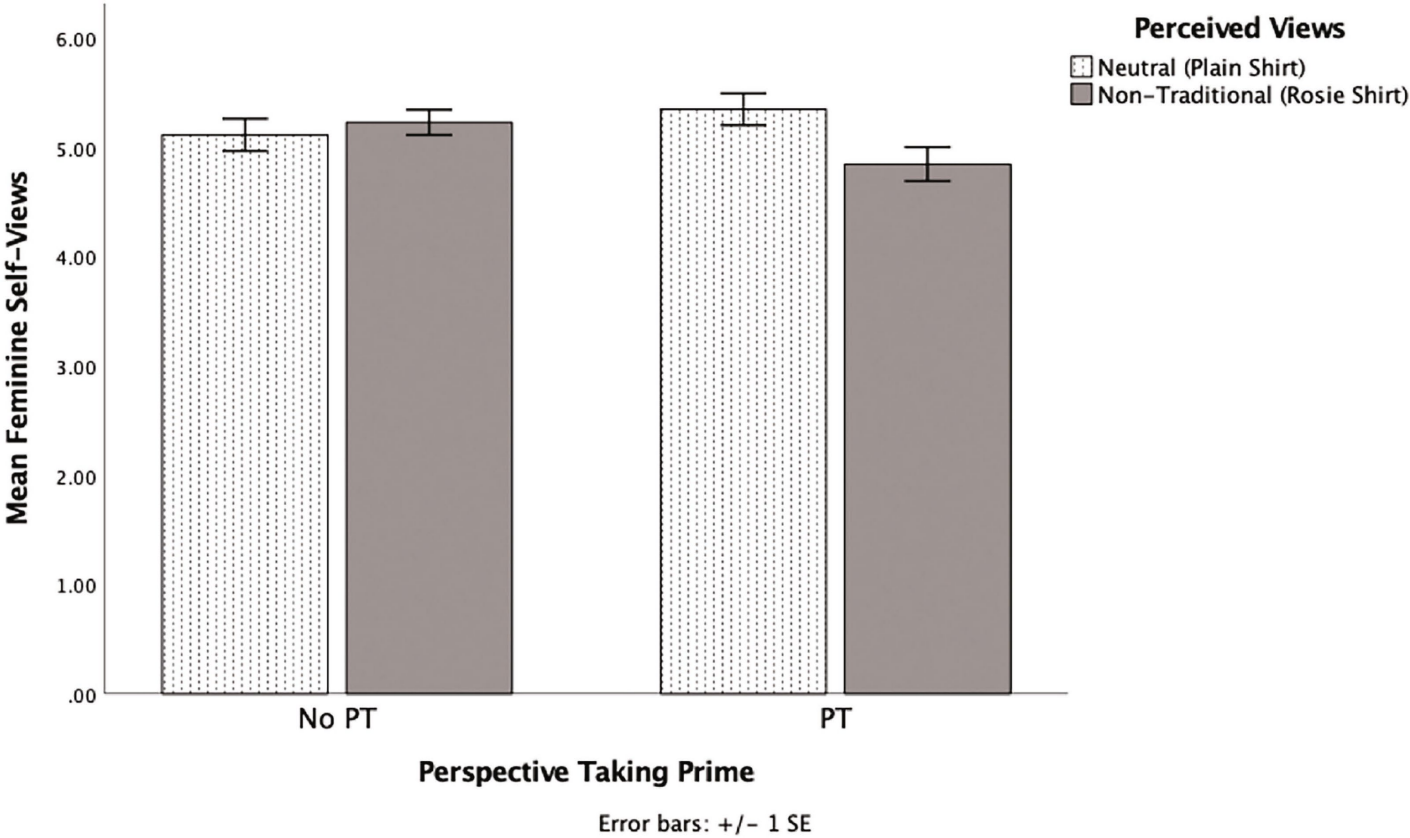
The effects of perspective taking and perceived views on feminine self-view in Experiment 2.

The analysis showed no statistically significant main effects for perspective taking (*p* = 0.599) or perceived views (*p* = 0.172). However, as predicted and seen in Figure 1, there was a statistically significant interaction between perspective taking and perceived views manipulations, *F* (1, 58) = 4.84, *p* = 0.032, *η^2^* = 0.077, two-tailed test. A simple effects analysis showed that perspective takers rated themselves as less feminine when the experimenter wore the Rosie t-shirt (*M* = 4.9, *SD* = 0.6) than when the experimenter wore the plain t-shirt (*M* = 5.4, *SD* = 0.6), *F* (1, 58) = 6.10, *p* = 0.017, *η^2^* = 0.095, 95% CI [0.10, 0.91], two-tailed test. This effect remained statistically significant when utilizing a bootstrap analysis with 1,000 samples (*p* = 0.02). Among those who interacted with the experimenter wearing the Rosie t-shirt, perspective takers (*M* = 4.9, *SD* = 0.6) rated themselves as less feminine than non-perspective takers (*M* = 5.2, *SD* = 0.5), *F* (1, 58) = 3.93, *p* = 0.052, *η^2^* = 0.064, 95% CI [−0.00, 0.77], two-tailed test. This pattern became marginal when conducting a bootstrap analysis with 1,000 samples (*p* = 0.06). There were no statistically significant differences for non-perspective takers (*p* = 0.556) or for those in the plain shirt condition (*p* = 0.254), and these effects were not statistically significant after bootstrapping with 1,000 samples. See Supplementary Table 8.

### Subliminal prime

A repeated measures ANOVA was conducted using the reaction times for “self” primed feminine positive words, “self” primed feminine negative words, “non-self” primed feminine positive words, and “non-self” primed feminine negative words as within variables and perspective taking and perceived views as between-participant factors (see Supplementary Table 9). There was no effect for the different traits (positive or negative; *p* = 0.156), nor were there any two-way (*p*s > 0.717) or three-way (*p* = 0.607) interactions.

We also conducted a repeated measures ANOVA using the reaction times for “self” primed masculine positive words, “self” primed masculine negative words, “non-self” primed masculine positive words, and “self” primed masculine negative words as within variables and the perspective taking prime and the perceived views as between participants factors. There were no statistically significant effects (*p*s > 0.232). See Supplementary Table 10.

### Perspective taking, affiliation and self-presentation

As in Experiments 1a and 1b, there were no effects on perspective taking (*p*s > 0.406), affiliation (*p*s > 0.376), or self-presentation (*p*s > 0.282). See Supplementary Table 11.

### Experiment 2 conclusion

The results support the hypothesis that perspective takers will also social tune when a partner holds gender nontraditional, or more feminist, views. In Experiment 2, perspective takers reported being less feminine than non-perspective takers when their partner wore a Rosie the Riveter t-shirt. However, this is only the case for explicit self-views, not implicit self-views.

## Experiment 3

The first three experiments show that perspective taking leads to social tuning for explicit self-views of women. However, it is unclear if these effects are limited to women and self-views or if they extend to implicit and explicit attitudes toward outgroup members as in past social tuning work (Sinclair et al., 2005b; Lun et al., 2007; Skorinko et al., 2015). While some past work shows that perspective taking reduces implicit and explicit biases (Galinsky and Moskowitz, 2000; Vescio et al., 2003; Dovidio et al., 2004; Galinsky et al., 2005, 2008; Shih et al., 2009, 2013; Todd et al., 2011; Todd and Burgmer, 2013; Gutierrez et al., 2014; Weyant, 2019), perspective taking may not always reduce biases (Galinsky and Ku, 2004; Vorauer et al., 2009; Paluck, 2010; Skorinko and Sinclair, 2013; Vorauer and Sasaki, 2014). Therefore, in Experiment 3, we investigate the role perspective taking plays in social tuning when the interaction partner endorses attitudes about an outgroup. We predict that perspective takers will be more likely to social tune explicit and implicit attitudes than non-perspective takers. However, the results of Experiment 2 make it less clear if perspective taking will lead to social tuning of implicit attitudes.

## Method

### Participants

One hundred and twenty-three participants (60 males; 63 females; 81 White, 5 Black, 20 Asian, 9 Latinx, 2 Middle Eastern, and 6 Multi-Racial) from a mid-Atlantic university and a medium-sized northeast private institution completed the experiment for course credit. Many (46%) were first year undergraduates (26% were second year; 10% third year; 16% fourth or fifth year undergraduates; 1% reported being a Graduate student; and 1% did not report). Since we are measuring attitudes toward Black individuals, five Black participants and two multi-racial participants who were part Jamaican were removed. Eight participants were removed for being an outlier or for an issue during their session (e.g., technology glitch, participant late, on their phone, etc.). Thus, the analyses are based off 109 participants (52 males; 57 females; 75 White, 19 Asian, 9 Latinx, 2 Middle Eastern, and 4 Multiracial).

### Design and materials

This experiment utilized a 2 (Perspective Taking Prime: Perspective Taking vs. No Perspective Taking) × 2 (Perceived Views: Egalitarian vs. Neutral Views) between-participants design.

#### Perspective taking manipulation

We used the same sentence unscrambling task used in Experiments 1a and 2.

#### Perceived views

The interaction partner was the experimenter. The experimenter was randomly assigned to wear a shirt that said “Eracism” or a plain shirt with no images/slogans, and all participants completed an ostensible eye test to ensure they read the message on the shirt.

#### Explicit racial attitudes

Participants indicated how strongly they agreed (1 = Strongly Disagree; 7 = Strongly Agree) with 5 items from the Modern Racism Scale (McConahay, 1986), 7 items from the Symbolic Racism Scale (Henry and Sears, 2002), and 1 item that appears on both scales. Five items on the Modern Racism Scale were recoded and four items on the Symbolic Racism Scale were recoded (see Appendix B for items). All 13 items were averaged together (Cronbach *α* = 0.89). Higher numbers indicate more egalitarian attitudes.

#### Implicit racial attitudes

To measure implicit racial views, we used the Black-White Implicit Association Task (IAT; Greenwald et al., 1998, 2003). In this IAT, participants categorize White and Black faces and “unpleasant” and “pleasant” words as quickly as possible. Using the recommended algorithm to compute the d-score (Nosek et al., 2005), the IAT measures how strongly individuals associate pleasant and unpleasant concepts with Black individuals. Higher positive d-scores indicate stronger positive implicit associations with Black individuals relative to White individuals.

#### Other measures

We used the same perceived views items but altered the language to be about valuing stereotypic or egalitarian people. We also used the same perspective taking (Cronbach *α* = 0.86), affiliation (Cronbach *α* = 0.71), and self-presentation (Cronbach *α* = 0.55) measures as in the previous studies. Participants reported demographic information (e.g., ethnicity/race, year in school, etc.).

### Procedure

Participants were greeted by an experimenter who was randomly assigned to wear a shirt that said “Eracism” (Egalitarian Views Condition) or a Plain shirt (No Views Condition). After providing informed consent, participants learned that the experiment examined cognitive skills and attitudes. Prior to completing any tasks, participants did an ostensible eye test to ensure they saw the messaging on the shirt. Participants then engaged in a cognitive skills task which was the perspective taking manipulation. After completing this task, the experimenter led the participants to a computer for the computer task, which was the Black-White IAT (Greenwald et al., 1998, 2003). The experimenter than provided the participant with a questionnaire that measured explicit attitudes, perspective taking, affiliation, self-presentation, and demographics. Participants placed their completed questionnaire in an envelope, sealed it, and placed it in a locked box. After putting the questionnaire in the box, the experimenter thanked the participant, debriefed them, and awarded credit.

## Results and discussion

### Perceived views manipulation check

A repeated measures ANOVA shows that participants believed the experimenter held more egalitarian (*M* = 4.3; *SD* = 1.8) than stereotypic (*M* = 2.5; *SD* = 1.3) views, *F* (1, 106) = 72.5, *p* < 0.001, *η^2^* = 0.406, two-tailed test. There was no interaction between the views with the perceived views condition (*p* = 0.601).

### Main analyses

We conducted a 2 (Perspective Taking: Perspective taking vs. None) x 2 (Perceived Views: Eracism t-shirt vs. Plain t-shirt) between-participants ANOVAs for the dependent measures.

### Explicit attitudes

There were no statistically significant main effects for perspective taking (*p* = 0.431), or perceived views (*p* = 0.693). However, there was a statistically significant interaction between perspective taking and perceived views, *F* (1, 105) = 4.02, *p* = 0.048, *η^2^* = 0.037, two-tailed test. Simple effects analyses showed that perspective takers who saw the Eracism t-shirt (*M* = 5.6, *SD* = 1.0) marginally rated themselves as more egalitarian than perspective takers who saw the plain t-shirt (*M* = 5.2, *SD* = 0.9), *F* (1, 105) = 2.85, *p* = 0.094, *η^2^* = 0.026, 95% CI [−0.07, 0.90], two-tailed test. This comparison remained marginal when a bootstrapped with 1,000 samples (*p* = 0.11). Of those who saw the Eracism t-shirt, perspective takers (*M* = 5.6, *SD* = 1.0) rated themselves as more egalitarian than non-perspective takers (*M* = 5.1, *SD* = 0.9), *F* (1, 105) = 3.86, *p* = 0.052, *η^2^* = 0.035, 95% CI [−0.00, 0.97], two-tailed test. This comparison became marginal when the data were bootstrapped for 1,000 samples (*p* = 0.06). However, no other comparisons were significant (*p*s > 0.255). See Supplementary Table 12.

### Implicit attitudes toward Black Individuals

There were no statistically significant main effects for perspective taking (*p* = 0.784), or perceived views (*p* = 0.705). There was also no statistically significant interaction between perspective taking and perceived views (*p* = 0.480). See Supplementary Table 13.

### Other measures

Contrary to Experiments 1a, 1b, and 2, perspective takers (*M* = 5.6; *SD* = 0.8) felt more affiliation toward the experimenter than non-perspective takers (*M* = 5.2, *SD* = 0.9), *F* (1, 105) = 5.24, *p* = 0.024, *η^2^* = 0.048, 95% CI [0.05, 0.69], two-tailed test. There was no statistically significant main effect for perceived views (*p* = 0.531) nor was there an interaction (*p* = 0.412) on affiliation. However, as in Experiments 1a, 1b, and 2, there were no statistically significant effects for perspective taking (*p*s > 0.271) or self-presentation (*p*s > 0.634). See Supplementary Table 14.

### Experiment 3 conclusion

Perspective takers were more likely to engage in social tuning and endorse more explicit egalitarian attitudes toward Black individuals than non-perspective takers. However, as in Experiment 2, perspective takers did not social tune their implicit attitudes. These results are not due to affiliation or self-presentation.

## Experiment 4

Thus far, perspective taking increases the likelihood of social tuning to the perceived views of an interaction partner than not perspective taking. However, unlike past social tuning work (Sinclair et al., 2005a,b; Lun et al., 2007; Skorinko et al., 2015), this alignment does not occur for implicit self-views or attitudes. In Experiment 4, we seek to understand why social tuning might occur for explicit, but not implicit attitudes. One possibility is that perspective taking highlights the perceived view of the partner, but also makes the perspective taker feel as if the partner expects them to be different. This may be heightened when the partner is wearing a shirt that endorses a specific viewpoint as a perspective taker might assume the partner is wearing the shirt because they believe others feel differently. Past work shows that when someone believes similar others view a stimulus (e.g., word, painting) in a different or distinct manner, it becomes less prominent in the mind (Shteynberg, 2010). This may explain why social tuning occurs for explicit but not implicit attitudes. Therefore, in Experiment 4, we explore whether the partner’s expectations influence perspective taking and social tuning. We predict that when the perceived views and expectations mismatch, perspective takers will social tune explicit but not implicit attitudes compared to when the views and expectations match.

## Method

### Participants

Ninety-five participants (72 Males, 23 Females, 1 Black, 11 Asian, 74 White, 2 Latinx, 5 Multiracial, and 2 Unreported) from a private northeast institution completed the experiment for course credit. Most were first (36%) or second year (27%) undergraduate students (14% third year; 22% fourth or fifth year; 1% were graduate students) Six participants who identified as Black or African, and six participants were removed for an issue occurring during their session (e.g., computer issue) or believing the Eracism shirt was racist. The analyses are based on 83 participants (62 Males, 21 Females; 69 White, 11 Asian, 2 Latinx, and 1 Other).

### Design and materials

This experiment utilized a 2 (Perspective Taking: Perspective Taking vs. No Perspective Taking) × 2 (Expectation: Egalitarian vs. Prejudiced) between-participants design. The experimenter always wore the “Eracism” t-shirt.

#### Stated expectations manipulation

Participants believed we were studying their social attitudes considering recent findings. Half the participants learned that the results of a recent study conducted by the Office of Diversity at their school found that students, on average, had unfavorable and stereotypic views toward different groups. This message was to elicit the expectation that the experimenter would assume participants were prejudiced, even though the experimenter endorsed egalitarian views by wearing an Eracism t-shirt. The other half of the participants learned that the results from this supposed study found that students, on average, had favorable and egalitarian views toward different groups. This messaged was to elicit the expectation that the experimenter would assume participants were egalitarian which matched their own views (i.e., Eracism t-shirt).

#### Perspective taking manipulation

We used the same sentence unscrambling task used in Experiments 1a, 2 and 3.

#### Explicit and implicit racial attitudes and other measures

We used the same explicit (Cronbach *α* = 0.77) and implicit measures as Experiment 3. We also used the same perceived views, perspective taking (Cronbach *α* = 0.83), affiliation (Cronbach *α* = 0.70), and self-presentation measures (Cronbach *α* = 0.65). Participants reported demographic information (e.g., ethnicity/race, year in school, etc.).

### Procedure

The experimenter greeted participants wearing an “Eracism” t-shirt. After giving informed consent, participants learned that we were investigating cognitive skill and attitudes. The experimenter explained that a recent study conducted at the university found that students were either more racially prejudiced or more egalitarian than expected. Thus, the experimenter’s own egalitarian views were either the same as their expectation of others or different. Participants than completed the vision test as in Experiments 2 and 3, and after finishing they unscrambled sentences for the perspective taking manipulation. The experimenter then took the participant to a computer to complete the Black-White IAT. After the IAT, participants completed the questionnaire that measured attitudes toward Black individuals, perspective taking, affiliation, self-presentation, and demographic information. After placing the questionnaire in an envelope and sealing it, participants were thanked, debriefed, and awarded credit.

## Results and discussion

### Manipulation check

A repeated measures ANOVA shows that participants believed the experimenter held more egalitarian (*M* = 4.0; *SD* = 1.4) than stereotypic (*M* = 2.9; *SD* = 1.4) views, *F* (1, 79) = 30.2, *p* < 0.001, *η*^2^ = 0.276, two-tailed test. There was no interaction between the stated expectation with the perceived views condition (*p* = 0.713).

### Main analyses

We conducted a 2 (Perspective Taking: Perspective taking vs. No Perspective Taking) x 2 (Stated Expectations: Prejudiced vs. Egalitarian) between-participants ANOVA.

### Explicit attitudes

**Figure 2:**
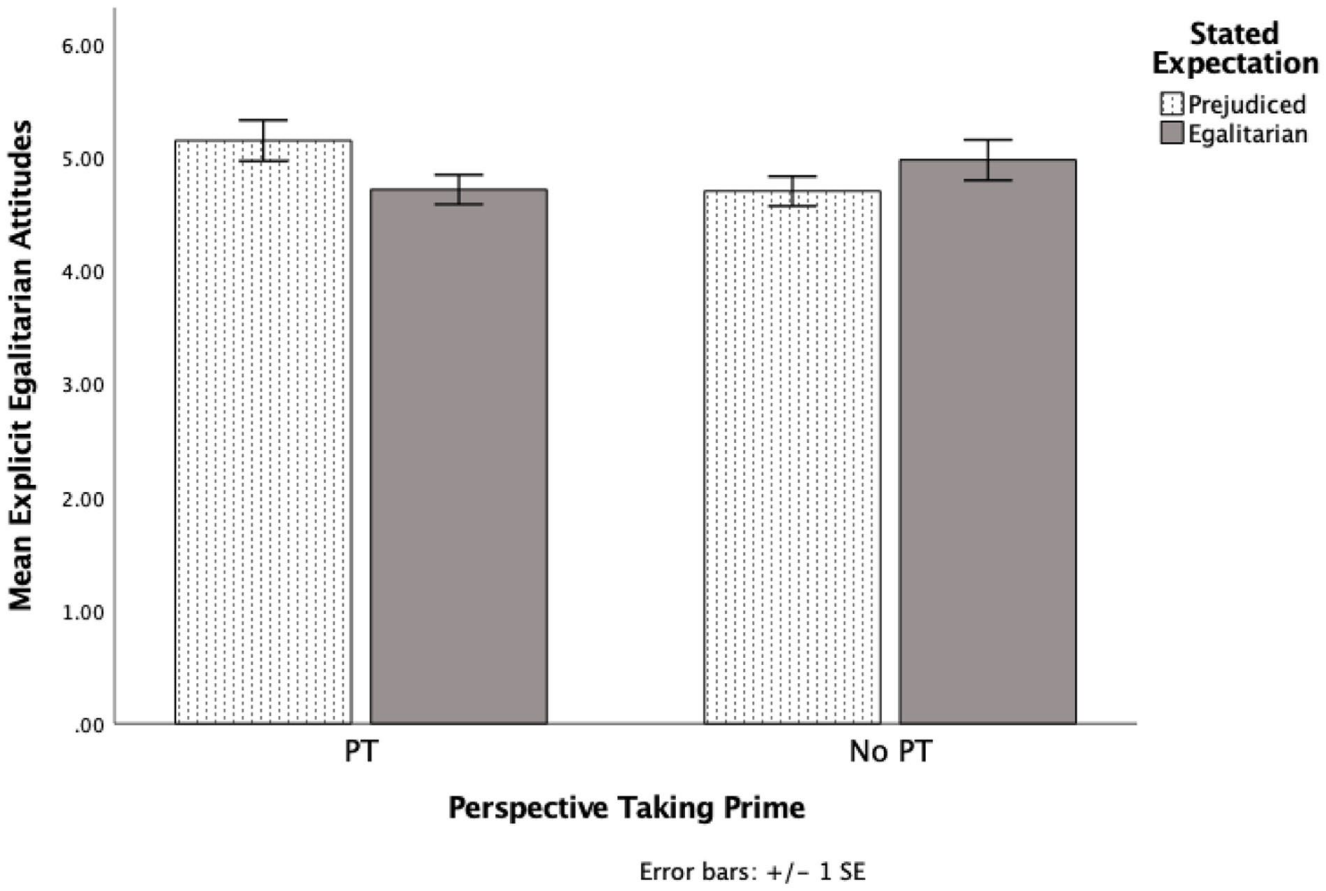
The effects of perspective taking and stated expectations on explicit attitudes toward Black individuals in Experiment 4.

There were no statistically significant main effects for perspective taking (*p* = 0.552) or stated expectations (*p* = 0.615). However, there was a statistically significant interaction between perspective taking and stated expectations, *F* (1, 79) = 5.17, *p* = 0.026, *η^2^* = 0.061, two-tailed test (see Figure 2). Simple effects analyses showed that as predicted, when expected to be prejudiced, perspective takers (*M* = 5.1, *SD* = 0.8) were more egalitarian than non-perspective takers (*M* = 4.7, *SD* = 0.6), *F* (1, 79) = 4.18, *p* = 0.044, *η^2^* = 0.050, 95% CI [0.01, 0.88], two-tailed test. This comparison remained statistically significant when bootstrapped with 1,000 samples (*p* = 0.05). Interestingly, perspective takers who were expected to be prejudiced (*M* = 5.1, *SD* = 0.8) were more egalitarian than perspective takers who were expected to be egalitarian (*M* = 4.7, *SD* = 0.6), *F* (1, 79) = 4.01, *p* = 0.049, *η^2^* = 0.048, 95% CI [0.00, 0.86], two-tailed test. This comparison became marginal bootstrapped with 1,000 samples (*p* = 0.055). However, there was no statistically significant effect when the stated expectation was to be egalitarian (*p* = 0.243) or for non-perspective takers (*p* = 0.223). See Supplementary Table 15.

### Implicit attitudes

Contrary to our predictions, there were no statistically significant main effects of perspective taking (*p* = 0.701) or the stated expectation (*p* = 0.557), nor was there an interaction (*p* = 0.629). See Supplementary Table 16.

### Other measures

There were no statistically significant effects for perspective taking (*p*s > 0.443), affiliation (*p*s > 0.440), or self-presentation (*p*s > 0.536). See Supplementary Table 17.

### Experiment 4 conclusion

Experiment 4 shows that perspective takers who knew their interaction partner expected them to be prejudiced social tuned with the perceived egalitarian views of the experimenter, not with the expectation. However, social tuning did not occur for implicit attitudes. Therefore, the differing beliefs and expectations of the partner did not prevent social tuning of explicit attitudes. Unexpectedly, perspective takers who learned their partner endorsed egalitarian beliefs and expected them to be egalitarian reported the same attitudes as non-perspective takers. It is possible that since the expectation to be egalitarian matched the perceived views that participants felt less of a need to social tune because there was already a shared reality.

## Experiment 5

Thus far, social tuning has not occurred for implicit attitudes for perspective takers—even when the partner’s beliefs and expectations are clear. In Experiment 5, we examine implicit attitudes and the role they play in social tuning when perspective taking. More specifically, we examine what happens when perspective takers are aware of their ostensible implicit attitudes at the beginning of an interaction. In Experiment 5, participants took the Black-White IAT and received ostensible results on the computer that revealed either positive or negative implicit views toward Black individuals. Participants then interacted with an experimenter who endorsed egalitarian views and were primed to perspective take (or not). We measured explicit and implicit attitudes (using a subliminal priming measure). We predicted perspective takers would social tune with perceived views of the partner regardless of the IAT results for explicit attitudes; however, we were unsure if implicit views would shift.

## Method

### Participants

Eighty-eight participants (48 Males, 37 Females, 3 No Report Gender, 7 Black, 9 Asian, 59 White, 5 Latinx, 4 Multiracial, and 4 Unreported) completed the experiment for course credit. Participants were evenly split across their year in school (30% first year; 17% second year; 25% third year; 25% fourth or fifth year; 3% unreported). Seven participants who identified as Black or African were removed, and six participants were removed for knowing the research assistant, not believing their IAT results, or doing a similar study. The analyses are based on 75 participants (41 Males, 31 Females, 3 No Report; 55 White, 8 Asian, 5 Latinx, 3 Multiracial, and 4 Did Not Report).

### Design and materials

This experiment utilized a 2 (Perspective Taking: Perspective Taking vs. No Perspective Taking) × 2 (Supposed IAT Result: Egalitarian vs. Prejudiced) between-participants design. The experimenter always wore the “Eracism” t-shirt.

#### Supposed IAT result

Participants believed they had to complete computer tasks to assess their cognitive skills. The first computer task they completed was a Black-White IAT. When they finished, the computer showed their results; however, these results were fake (and randomly assigned). Half the participants received IAT scores indicating they associated White with pleasant (e.g., more implicit prejudice) and the other half received scores indicating they associated Black individuals with Pleasant (e.g., less implicit prejudice). After reviewing their score, participants closed out the program themselves. It was clear that the experimenter did not know their results.

#### Perspective taking manipulation

We used the same sentence unscrambling task used in Experiments 1a, 2, 3, and 4.

#### Explicit and implicit racial attitudes

We used the same explicit measure as Experiments 3 and 4 (Cronbach *α* = 0.88). However, since we used the Black-White IAT to give fake feedback, we measured implicit attitudes with a subliminal priming measure. As in Experiment 2, participants kept their eyes fixated on a dot in the middle of the screen, and quickly indicated as fast as possible if the word that appeared in the middle of the screen was “good” by pressing the “S” key or “bad” by pressing the “L” key. Prior to a stimulus appearing in the middle of the screen, participants saw an image of a sunflower in one of the four quadrants on the screen. This is how they were primed with either a white face or a black face. Each prime was forward masked with the sunflower image for 300 ms, the White or Black face prime showed for 15 ms, and then the prime was backward masked with the sunflower image for 300 ms. The latencies for each stimulus were log transformed, and then the logged latencies for each category were averaged together.

#### Other measures

We also used the same perceived views, perspective taking (Cronbach *α* = 0.86), affiliation (Cronbach *α* = 0.71), and self-presentation measures (Cronbach *α* = 0.61). Participants reported demographic information (e.g., ethnicity/race, year in school, etc.).

### Procedure

A white experimenter wearing an “Eracism” shirt, greeted participants and explained that the study investigated cognitive skills and attitudes toward different social groups. After giving informed consent, participants completed an ostensible eye test to make sure they read the message on the experimenter’s shirt. After the vision test, they completed the first “cognitive task.” This was counterbalanced to be either the perspective taking sentence unscrambling task or the Black-White IAT. As soon as participants completed the IAT, the computer auto-generated their score, but we rigged the score. Half the participants learned that their IAT results revealed that they were more favorable to White individuals (e.g., more implicit prejudice), and the other half learned that their results showed that they were more favorable to Black individuals (e.g., less implicit prejudice). Participants then completed the explicit measure and implicit subliminal prime measure which were also counterbalanced. Participants placed their questionnaire in an enveloped and sealed it. After completing all the measures, participants were thanked, debriefed, and awarded credit.

## Results and discussion

### Perceived views manipulation check

A repeated measures ANOVA shows that participants believed the experimenter held more egalitarian (*M* = 4.4; *SD* = 1.4) than stereotypic (*M* = 2.6; *SD* = 1.3) views, *F* (1, 71) = 44.8, *p* < 0.001, *η^2^* = 0.387, two-tailed test. There was no interaction between the stated expectation with the perceived views condition (*p* = 0.655).

### Main analyses

We conducted a 2 (Perspective Taking: Perspective taking vs. Non-Perspective taking) × 2 (IAT Result: Prejudiced vs. Egalitarian) between-participants ANOVA on the dependent measures. The order in which participants received the IVs and DVs were covariates.

### Explicit attitudes

There were no statistically significant main effects for perspective taking (*p* = 0.159) or IAT result (*p* = 0.428). However, there was a statistically significant interaction between perspective taking prime and IAT result, *F* (1, 69) = 4.42, *p* = 0.039, *η^2^* = 0.060, two-tailed test. Simple effects analyses showed when receiving IAT results indicating more implicit prejudice, perspective takers (*M* = 4.72, *SD* = 0.80) had *more* egalitarian attitudes than non-perspective takers (*M* = 4.02, *SD* = 1.30), *F* (1, 69) = 6.23, *p* = 0.015, *η^2^* = 0.083, 95% CI [0.15, 1.36], two-tailed test. The finding remained statistically significant when bootstrapped with 1,000 samples (*p* = 0.05). There were no differences for those receiving egalitarian IAT results (*p* = 0.630). Perspective takers reported similar egalitarian attitudes regardless of if their ostensible IAT results (*p* = 0.391). But non-perspective takers with more implicit prejudice IAT results (*M* = 4.03, *SD* = 1.30) reported *less* egalitarian attitudes than non-perspective takers with more egalitarian implicit attitudes (*M* = 4.65, *SD* = 0.88), *F* (1, 69) = 4.38, *p* = 0.040, *η^2^* = 0.060, 95% CI [0.03, 1.23], two-tailed test. The finding became marginal when bootstrapped with 1,000 samples (*p* = 0.09). See Supplementary Table 18.

### Implicit subliminal priming task and other measures

There were no statistically significant main effects of perspective taking (*p* = 0.904) or IAT Results (*p* = 0.296), nor was there a statistically significant interaction (*p* = 0.375) on implicit attitudes (see Supplementary Table 19). There were also no effects for perspective taking (*p*s > 0.320) or self-presentation (*p*s > 0.149). See Supplementary Table 20.

While there were no statistically significant main effects of perspective taking (*p* = 0.690) or IAT Results (*p* = 0.595) for affiliation; there was an interaction, *F* (1, 69) = 5.3, *p* = 0.024, *η^2^* = 0.073, two-tailed test. The only simple effect that was statistically significant was for non-perspective takers. Those with prejudiced IAT results (*M* = 4.3; *SD* = 1.0) reported *less* affiliation with the experimenter than those with egalitarian IAT results (*M* = 5.0; *SD* = 0.9), *F* (1, 69) = 4.02, *p* = 0.049, 95% CI [0.00, 1.31], *η^2^* = 0.057, two-tailed test, with a medium effect. This result became marginal when bootstrapped with 1,000 samples (*p* = 0.07). See Supplementary Table 20.

### Experiment 5 conclusion

When participants believed they had more implicit prejudice, perspective takers were more likely to social tune with the perceived egalitarian views of the experimenter than non-perspective takers, but only for explicit and not implicit attitudes. But, when participants believed they had more egalitarian implicit views, perspective takers and non-perspective takers did not differ in their explicit or implicit views. As in Experiment 4, it appears that actively realizing there were shared beliefs with the partner resulted in less of a need to social tune.

## General discussion

Across six experiments, perspective takers were more likely to engage in social tuning and align their self-views and explicit racial attitudes with the perceived views of an ostensible interaction partner than non-perspective takers. This happened even when it resulted in self-stereotyping (Experiments 1a and 1b). However, perspective takers never social tuned their implicit attitudes with the perceived views of their ostensible partner (Experiments 2, 3, 4, 5). This finding is inconsistent with past work that has found social tuning of implicit views (Lun et al., 2007; Skorinko and Sinclair, 2018) and past work showing that perspective taking reduced implicit prejudice (Todd et al., 2011; Shih et al., 2013; Gutierrez et al., 2014; Todd and Galinsky, 2014; Weyant, 2019). However, it does align with studies that have found that perspective taking may not change or reduce implicit bias (Aberson and Haag, 2007; Todd and Simpson, 2016; Edwards et al., 2017), or that implicit attitudes may not change in the same way as explicit attitudes (Forscher et al., 2019).

The reason for the lack of social tuning on implicit attitudes for perspective takers is still unclear. We tested the hypothesis that perspective takers believe that their partner might endorse a view but expected them to feel differently (especially when wearing a shirt endorsing a particular view) and this distinction (Shteynberg, 2010) limited social tuning for implicit views. However, even when the experimenter’s expectations clearly matched or mismatched the perceived views, perspective takers did not social tune their implicit attitudes (Experiment 4). We also examined whether knowing one’s (ostensible) implicit views might influence perspective taking and social tuning of implicit views. But, once again, perspective takers did not social tune their implicit attitudes (Experiment 5). The lack of social tuning on implicit attitudes does not appear to be related to perspective takers assumptions of what their interaction partner expects of them, or how different they might feel with their partner (Shteynberg, 2010; Hodges et al., 2018). One key difference is that in previous studies perspective takers put themselves in the shoes of an outgroup member (Todd et al., 2011; Shih et al., 2013; Gutierrez et al., 2014; Todd and Galinsky, 2014; Weyant, 2019); whereas in the current work, participants perspective taking target was not an outgroup member. Thus, future work may examine who the perspective taking target is and if that influences the social tuning of implicit attitudes. Future work could also investigate other factors such as the natural tendency to perspective (IRI; Davis, 1980), cognitive load, or even accuracy (Nickerson, 1999).

Perspective taking is also presumed to be an important component in the fulfillment of affiliative needs (Hardin and Conley, 2001; Sinclair et al., 2005a). However, we found very limited evidence that perspective taking influenced affiliation. While perspective takers felt more affiliation with their interaction partner than non-perspective takers in Experiment 3, this did not replicate. Perspective taking also led to social tuning with or without affiliative motivation (Experiment 1b). One possible explanation is that perspective taking is important for picking up on the perceived views of the interaction partner, but empathy, which is more focused on how someone else feels, may be needed to elicit more affiliation. It is possible the lack of affiliation contributes to the lack of social tuning for implicit attitudes. More research is needed to understand the link, if any, between affiliation, perspective taking, and empathy in social tuning and shared reality.

These experiments have a few limitations. Perspective taking is often manipulated by instructing a participant to engage in it (e.g., through a day in the life essay, watching a movie). However, this seemed less likely in a real-world situation so we utilized a more subtle manipulation of perspective taking using mindset priming procedures (e.g., a sentence unscrambling task, a mindset prime). It is possible that this subtle manipulation was less likely to influence social tuning of implicit views compared to other, more direct, manipulations. Future work should explore this further.

Likewise, perspective takers did not report perspective taking more with their partner. While this may appear to be a failure of the manipulation, the mindset tasks were pretested and induced perspective taking. Also, given the subtle nature of the manipulation, we did not anticipate that participants would be able to consciously articulate their perspective taking actions. This is consistent with past work where participants were not conscious or able to report goals from sentence unscrambling tasks (Ferguson, 2008). It is also possible that the perspective taking items were administered too late for accurate reporting (Hauser et al., 2018). Relatedly, we only investigated natural perspective taking tendencies such as those measured by the Interpersonal Reactivity Index (IRI, Davis, 1980) in Experiment 1a and found no effects. Future research could explore whether those who score highly on perspective taking tendences are more likely to social tune, especially for implicit attitudes, or if a more specific strategy to harness that natural tendency is needed like an implementation intention (Mendoza et al., 2010).

An additional limitation of the current work may be the sample sizes, especially when manipulating perspective taking (Huang et al., 2021). *A priori* power analyses were unable to be conducted as no work has directly examined perspective taking and social tuning. We collected as many participants as was logistically possible given resource constraints for each experiment (see Laken, 2022). To try to rule out the possibility that any statistically significant findings were not a Type 1 error, we conducted six experiments to confirm the existence of our key findings. Over the six experiments, we demonstrate a proof of concept for an effect of perspective taking on the social tuning of explicit, but not implicit, self-views and attitudes. We report effect sizes for future researchers to conduct *a priori* power analyses and recommend larger sample sizes when within the resource constraints of researchers.

In conclusion, the results of six experiments show that perspective taking can lead to social tuning of self-views and explicit attitudes, but not implicit attitudes. As in past work, these results are not due to self-presentation, at least based on the restrictive view (Sinclair et al., 2005a). Thus, the current research adds to our knowledge of different factors that lead to social tuning. It also adds to existing work that finds that that perspective taking may not readily influence or change implicit attitudes (Aberson and Haag, 2007; Todd and Simpson, 2016; Edwards et al., 2017). While perspective taking contributes to the transmission of attitudes, future work needs to further examine the role of perspective taking in shared reality as it appears to be as complicated as Hodges et al. (2018) argue. In conclusion, perspective taking leads to social tuning of explicit views, but appears to be an inhibitor for social tuning of implicit views.

## Data availability statement

The raw data supporting the conclusions of this article will be made available by the authors, without undue reservation.

## Ethics statement

The studies involving human participants were reviewed and approved by Worcester Polytechnic Institution. The patients/participants provided their written informed consent to participate in this study.

## Author contributions

JS developed the idea, helped design the experiments, advised the student co-authors, analyzed the results, and wrote the majority of the manuscript. CD, KR, AT, JS, MK, LL, DV, and PB all contributed equally to the work. CD, MK, LL, DV, and PB assisted with conducting and analyzing Experiment 3. JS helped design and conduct Experiment 1b. KR and AT helped conduct and analyze Experiment 1b. M-SJ collaborated and co-advised the work conducted on Experiment 1b and contributed to the writing of the manuscript. AD contributed to the writing of the manuscript. All authors contributed to the article and approved the submitted version.

## Conflict of interest

The authors declare that the research was conducted in the absence of any commercial or financial relationships that could be construed as a potential conflict of interest.

## Publisher’s note

All claims expressed in this article are solely those of the authors and do not necessarily represent those of their affiliated organizations, or those of the publisher, the editors and the reviewers. Any product that may be evaluated in this article, or claim that may be made by its manufacturer, is not guaranteed or endorsed by the publisher.
