# Supplementary material for: The effects of perspective taking primes on the social tuning of explicit and implicit views toward gender and race
Source: Front Psychol. 2023 Mar 2;14:1014803. doi: 10.3389/fpsyg.2023.1014803 (PMC10020926; doi:10.3389/fpsyg.2023.1014803)
Supplement: Supplementary file 1 [file Data_Sheet_1.docx]

**Appendix A**

**Perspective Taking Subscale Interpersonal Reactivity Index (IRI; Davis, 1980)**

1. I sometimes find it difficult to see things from the “other guy’s” point of view. (Recoded)
2. I try to look at everybody’s side of a disagreement before I make a decision.
3. I sometimes try to understand my friends better by imagining how things look from their perspective.
4. If I’m sure I’m right about something, I don’t waste much time listening to other people’s arguments. (Recoded)
5. I believe there are two sides to every question and try to look at them both.
6. When I’m upset at someone, I usually try to “put myself in his shoes” for a while.

**Perspective Taking Measure**

1. How motivated are you to put yourself in your partner’s shoes?
2. How important is it for you to try to think about yourself from your partner’s standpoint?
3. To what extend are you able to see the world through your partner’s eyes?
4. How easily were you able to take the perspective of your partner?
5. How able were you to understand your partner’s standpoint?

**Appendix B**

**Items Used in the Explicit Attitudes Measure in Experiments 3, 4, and 5**

**Modern Racism Scale (McConahay, 1986)**

1. Over the past few years, the government and news media have shown more respect for blacks than they deserve. (Recoded)
2. It is easy to understand the anger of black people in America.
3. Blacks are getting too demanding in their push for equal rights. (Recoded)
4. Discrimination against blacks is no longer a problem in the United States. (Recoded)
5. Blacks should not push where they are not wanted. (Recoded)

**Symbolic Racism Scale (Henry & Sears, 2002)**

1. It’s really a matter of some people not trying hard enough; if blacks would only try harder, they could be just as well off as whites. (Recoded)
2. Irish, Italian, Jewish, and many other minorities overcame prejudice and worked their way up. Blacks should do the same. (Recoded)
3. Black leaders have been trying to push too fast. (Recoded)
4. How much of the racial tension that exists in the United States today do you think blacks are responsible for creating? (Recoded)
5. How much discrimination against blacks do you feel there is in the United States today, limiting their chances to get ahead?
6. Generations of slavery and discrimination have created conditions that make it difficult for blacks to work their way out of the lower class.
7. Over the past few years, blacks have gotten less than they deserve.

**From Both Scales**

1. Over the past few years, blacks have gotten more economically than they deserve. (Recoded)
