# Supplementary material for: The effects of perspective taking primes on the social tuning of explicit and implicit views toward gender and race
Source: Front Psychol. 2023 Mar 2;14:1014803. doi: 10.3389/fpsyg.2023.1014803 (PMC10020926; doi:10.3389/fpsyg.2023.1014803)
Supplement: Supplementary file 2 [file Data_Sheet_2.docx]

**Supplemental Materials for** **“The Effects of Perspective Taking Primes on the Social Tuning of Explicit and Implicit Views Towards Gender and Race”**

**Table of Contents**

Table 1: Feminine Ratings in Experiment 1a 2

Table 2: Self-Other Overlap in Experiment 1a 3

Table 3: PT (IRI), Partner PT, Affiliation, and Self-Presentation in Experiment 1a 4

Table 4: Feminine Ratings in Experiment 1b 5

Table 5: Self-Other Overlap in Experiment 1b 6

Table 6: PT Partner, Affiliation, and Self-Presentation in Experiment 1b 7

Table 7: Perceived Views Manipulation Check in Experiment 2 8

Table 8: Feminine Ratings in Experiment 2 9

Table 9: Implicit Feminine Ratings in Experiment 2 10

Table 10: Implicit Masculine Ratings in Experiment 2 11

Table 11: PT Partner, Affiliation, and Self-Presentation in Experiment 2 12

Table 12: Explicit Egalitarian Views in Experiment 3 13

Table 13: Implicit Egalitarian Views in Experiment 3 14

Table 14: PT Partner, Affiliation, and Self-Presentation in Experiment 3 15

Table 15: Explicit Egalitarian Views in Experiment 4 16

Table 16: Implicit Egalitarian Views in Experiment 4 17

Table 17: PT Partner, Affiliation and Self-Presentation in Experiment 4 18

Table 18: Explicit Egalitarian Views in Experiment 5 19

Table 19: Implicit Egalitarian Views in Experiment 5 20

Table 20: PT Partner, Affiliation, and Self-Presentation in Experiment 5 21

**Table 1**

*Descriptive and Inferential Statistics for Perspective Taking on Feminine Trait Ratings in Experiment 1a*

| Condition | *N* | *M* | *SD* | Min | Max | Skew | *F* | *p* | *η^2^* | *CI*s |
| --- | --- | --- | --- | --- | --- | --- | --- | --- | --- | --- |
| Perspective Taking |  |  |  |  |  |  | 4.25 | .045^*^ | .092 | .01, .71 |
| PT | 14 | 4.9 | .4 | 4.4 | 5.6 | .40 |  |  |  |  |
| No PT | 30 | 4.5 | .6 | 2.8 | 5.7 | -.74 |  |  |  |  |

*Note.* * = *p* ≤.05. The analysis was a one-way ANOVA with a two-tailed test. 95% Confidence Intervals are reported.

**Table 2**

*Descriptive and Inferential Statistics for Perspective Taking on Self-Other Overlap of Feminine Ratings in Experiment 1a*

| Comparison | | *N* | *M* | *SD* | *F* | *p* | *η^2^* | *CI*s |
| --- | --- | --- | --- | --- | --- | --- | --- | --- |
| Ratings | |  |  |  | .67 | .419 | .016 | -.09, .22 |
| Self | | 44 | 4.6 | .6 |  |  |  |  |
| Partner | | 44 | 4.6 | .6 |  |  |  |  |
|  | |  |  |  |  |  |  |  |
| PT * Ratings | |  |  |  | .30 | .587 | .007 |  |
| PT Self | | 14 | 4.9 | .4 |  |  |  |  |
| NoPT Self | | 30 | 4.5 | .6 |  |  |  |  |
| PT Partner  NoPT Partner | | 14  30 | 4.8  4.5 | .4  .8 |  |  |  |  |
| Self: PT v NoPT | |  |  |  | 4.25 | .045^*^ | .092 | .01, .71 |
| Partner: PT v NoPT | |  |  |  | 1.38 | .25 | .032 | -.19, .74 |
| PT: Self v Partner | |  |  |  | .68 | 413 | .016 | -.15, .37 |
| NoPT: Self v Partner | |  |  |  | .06 | .813 | .001 | -.16, .20 |
|  |  | |  |  |  |  |  |  |

*Note.* * = *p* ≤.05. The analysis was a repeated measures ANOVA with a two-tailed test. 95% Confidence Intervals are reported.

**Table 3**

*Descriptive and Inferential Statistics for Perspective Taking on Interpersonal Reactivity Index (IRI) Perspective Taking Subscale, Perspective Taking with Partner, Affiliation, and Self-Presentation in Experiment 1a*

| DV  Comparison | *N* | *M* | *SD* | *F* | *p* | *η^2^* | *CI*s |
| --- | --- | --- | --- | --- | --- | --- | --- |
| **IRI (PT)** |  |  |  | .19 | .663 | .005 | -.88, .57 |
| PT | 9 | 4.6 | .5 |  |  |  |  |
| Control | 28 | 4.8 | 1.0 |  |  |  |  |
|  |  |  |  |  |  |  |  |
| **PT Measure** |  |  |  | .99 | .325 | .023 | -.77, .26 |
| PT | 14 | 3.6 | 1.0 |  |  |  |  |
| Control | 30 | 3.9 | .7 |  |  |  |  |
|  |  |  |  |  |  |  |  |
| **Affiliation** |  |  |  | 3.4 | 074 | .074 | -1.2, .06 |
| PT | 14 | 4.1 | 1.1 |  |  |  |  |
| Control | 30 | 4.7 | .7 |  |  |  |  |
|  |  |  |  |  |  |  |  |
| **Self-Presentation** |  |  |  | .23 | .632 | .006 | -.94, .58 |
| PT | 14 | 3.8 | 1.4 |  |  |  |  |
| Control | 30 | 4.0 | 1.0 |  |  |  |  |

*Note.* * = *p* ≤.05. The analyses were one-way ANOVAs with a two-tailed test. 95% Confidence Intervals are reported.

**Table 4**

*Descriptive and Inferential Statistics for Perspective Taking and Affiliative Motivation on Feminine Ratings in Experiment 1b*

| Comparison | | *N* | *M* | *SD* | Min | Max | Skew | *F* | *p* | *η^2^* | *CI*s |
| --- | --- | --- | --- | --- | --- | --- | --- | --- | --- | --- | --- |
| PT | |  |  |  |  |  |  | 9.02 | .004^*^ | .119 | -.76, -.15 |
| PT | | 37 | 5.2 | .8 | 4.0 | 6.4 | 1.0 |  |  |  |  |
| No PT | | 34 | 4.8 | .6 | 2.8 | 5.8 | -1.0 |  |  |  |  |
|  | |  |  |  |  |  |  |  |  |  |  |
| Affiliation | |  |  |  |  |  |  | .07 | .800 | .001 | -.27, .34 |
| AM | | 33 | 5.0 | .6 | 3.5 | 6.0 | -.51 |  |  |  |  |
| No AM | | 38 | 5.0 | .7 | 2.8 | 6.4 | .04 |  |  |  |  |
|  | |  |  |  |  |  |  |  |  |  |  |
| PT * AM | |  |  |  |  |  |  | .33 | .565 | .005 |  |
| NoPT, NoAM | | 17 | 4.7 | .7 | 2.8 | 5.8 | -1.2 |  |  |  |  |
| NoPT, AM | | 17 | 4.8 | .5 | 3.5 | 5.8 | -.44 |  |  |  |  |
| PT, NoAM | | 21 | 5.2 | .7 | 4.3 | 5.3 | .28 |  |  |  |  |
| PT, AM | | 16 | 5.1 | .7 | 4.0 | 6.0 | -.11 |  |  |  |  |
| NoAM: PT v No | |  |  |  |  |  |  | 6.86 | .011^*^ | .093 | .13, .96 |
| AM: PT v No | |  |  |  |  |  |  | 2.76 | .101 | .040 | -.07, .82 |
| NoPT: AM v No | |  |  |  |  |  |  | .05 | .823 | .001 | -.49, .39 |
| PT: AM v No | |  |  |  |  |  |  | .36 | .551 | .005 | -.30, .55 |
|  | |  |  |  |  |  |  |  |  |  |  |
| PT Conditions | |  |  |  |  |  |  | 3.25 | .047^*^ | .113 |  |
|  | |  |  |  |  |  |  | *t* | *p* |  | *CIs* |
| None v PTNoAM | |  |  |  |  |  |  | 2.48 | .016 |  | .10, .99 |
| None v PTAM | |  |  |  |  |  |  | 1.78 | .081 |  | -.05, 89 |
| PTNoAM v PTAM | |  |  |  |  |  |  | .57 | .573 |  | -.58, .32 |
|  |  | |  |  |  |  |  |  |  |  |  |

*Note.* * = *p* ≤.05. The analysis was a two-way ANOVA with a two-tailed test. 95% Confidence Intervals are reported. This also includes the one-way ANOVA that was conducted comparing the No Perspective Taking and No Affiliative Motivation (None) condition with the Perspective Taking and No Affiliative Motivation (PTNoAM) Condition and the Perspective Taking and Affiliative Motivation (PTAM) Condition.

**Table 5**

*Descriptive and Inferential Statistics for Perspective Taking and Affiliative Motivation on Self-Other Overlap of Feminine Ratings in Experiment 1b*

| Comparison | *N* | *M* | *SD* | *F* | *p* | *η^2^* | *CI*s |
| --- | --- | --- | --- | --- | --- | --- | --- |
| Ratings |  |  |  | 86.5 | .001 | .567 | -.09, .22 |
| Self | 44 | 4.6 | .60 |  |  |  |  |
| Partner | 44 | 4.6 | .55 |  |  |  |  |
|  |  |  |  |  |  |  |  |
| PT * Ratings |  |  |  | 3.03 | .087 | .044 |  |
| PT Self | 36 | 5.2 | .65 |  |  |  |  |
| PT Partner | 36 | 3.9 | .63 |  |  |  |  |
| NoPT Self | 34 | 4.7 | .73 |  |  |  |  |
| NoPT Partner | 34 | 3.9 | .73 |  |  |  |  |
|  |  |  |  |  |  |  |  |
| AM * Rating |  |  |  | .395 | .532 | .006 |  |
| PT *AM * Rate |  |  |  | .030 | .862 | .000 |  |
|  |  |  |  |  |  |  |  |

*Note.* * = *p* ≤.05. The test was a repeated measures ANOVA two-tailed test. 95% Confidence Intervals are reported.

**Table 6**

*Descriptive and Inferential Statistics for Perspective Taking and Affiliative Motivation on Perspective Taking, Affiliation, and Self-Presentation in Experiment 1b*

| DV  Comparison | *N* | *M* | *SD* | *F* | *p* | *η^2^* | *CI*s |
| --- | --- | --- | --- | --- | --- | --- | --- |
| **PT Measure** |  |  |  |  |  |  |  |
| PT |  |  |  | .270 | .605 | .004 | -.80, .47 |
| PT | 36 | 4.0 | 1.4 |  |  |  |  |
| Control | 34 | 3.9 | 1.3 |  |  |  |  |
|  |  |  |  |  |  |  |  |
| AM |  |  |  | 1.35 | .249 | .020 | -1.0, .27 |
| AM | 38 | 4.2 | 1.3 |  |  |  |  |
| Control | 32 | 3.8 | 1.3 |  |  |  |  |
|  |  |  |  |  |  |  |  |
| PT*AM |  |  |  | .467 | .497 | .007 |  |
| PT/AM | 15 | 4.4 | 1.3 |  |  |  |  |
| PT/NoAM | 21 | 3.8 | 1.4 |  |  |  |  |
| NoPT/AM | 17 | 4.0 | 1.4 |  |  |  |  |
| None | 17 | 3.8 | 1.2 |  |  |  |  |
|  |  |  |  |  |  |  |  |
| **AM Measure** |  |  |  |  |  |  |  |
| AM |  |  |  | .94 | .336 | .014 | -.96, .33 |
| AM | 32 | 3.7 | 1.4 |  |  |  |  |
| Control | 28 | 3.4 | 1.3 |  |  |  |  |
|  |  |  |  |  |  |  |  |
| PT |  |  |  | .00 | .975 | .000 | -.65, .63 |
| PT | 36 | 3.6 | 1.4 |  |  |  |  |
| Control | 34 | 3.6 | 1.2 |  |  |  |  |
|  |  |  |  |  |  |  |  |
| PT*AM |  |  |  | .94 | .336 | .014 |  |
| PT/AM | 15 | 3.9 | 1.6 |  |  |  |  |
| PT/NoAM | 21 | 3.3 | 1.3 |  |  |  |  |
| NoPT/AM | 17 | 3.6 | 1.3 |  |  |  |  |
| None | 17 | 3.6 | 1.2 |  |  |  |  |
|  |  |  |  |  |  |  |  |
| **Self-Present** |  |  |  |  |  |  |  |
| PT |  |  |  | .02 | .878 | .000 | -.64, .75 |
| PT | 36 | 3.2 | 1.5 |  |  |  |  |
| Control | 34 | 2.2 | 1.4 |  |  |  |  |
|  |  |  |  |  |  |  |  |
| AM |  |  |  | .00 | .965 | .000 | -.68, .71 |
| AM | 32 | 3.3 | 1.4 |  |  |  |  |
| Control | 38 | 3.3 | 1.5 |  |  |  |  |
|  |  |  |  |  |  |  |  |
| PT*AM |  |  |  | .10 | .766 | .001 |  |
| PT/AM | 15 | 3.2 | 1.4 |  |  |  |  |
| PT/NoAM | 21 | 3.3 | 1.6 |  |  |  |  |
| NoPT/AM | 17 | 3.3 | 1.4 |  |  |  |  |
| None | 17 | 3.2 | 1.3 |  |  |  |  |
|  |  |  |  |  |  |  |  |

*Note.* * = *p* ≤.05. The test was a repeated measures ANOVA two-tailed test. 95% Confidence Intervals are reported.

**Table 7**

*Descriptive and Inferential Statistics for Extent Partner Valued Gender Traditional compared to Nontraditional People by the Perceived Views Manipulation in Experiment 2*

| Comparison | *N* | *M* | *SD* | *F* | *p* | *η^2^* | *CI*s |
| --- | --- | --- | --- | --- | --- | --- | --- |
| Ratings |  |  |  | .99 | .323 | .016 | -.53, .18 |
| Trad | 62 | 4.2 | 1.2 |  |  |  |  |
| NonTrad | 62 | 4.3 | 1.1 |  |  |  |  |
|  |  |  |  |  |  |  |  |
| Views * Trad |  |  |  | .00 | .979 | .000 |  |
| Trad Plain | 29 | 4.2 | 1.2 |  |  |  |  |
| Trad Rosie | 33 | 4.1 | 1.2 |  |  |  |  |
| NTrad Plain  NTrad Rosie | 29  33 | 4.4  4.2 | .8  1.2 |  |  |  |  |
|  |  |  |  |  |  |  |  |

*Note.* * = *p* ≤.05. The test was a repeated measures ANOVA two-tailed test. 95% Confidence Intervals are reported.

**Table 8**

*Descriptive and Inferential Statistics for Perspective Taking and Perceived Views on Feminine Ratings in Experiment 2*

| Comparison | *N* | *M* | *SD* | Min | Max | Skew | *F* | *p* | *η^2^* | *CI*s |
| --- | --- | --- | --- | --- | --- | --- | --- | --- | --- | --- |
| PT |  |  |  |  |  |  | .28 | .599 | .005 | -.21, .35 |
| PT | 29 | 5.1 | .6 | 3.6 | 6.0 | .41 |  |  |  |  |
| No PT | 33 | 5.2 | .5 | 4.3 | 6.5 | -.38 |  |  |  |  |
|  |  |  |  |  |  |  |  |  |  |  |
| Views |  |  |  |  |  |  | 1.9 | .172 | .032 | -.09, .47 |
| Rosie | 33 | 5.0 | .6 | 3.6 | 6.5 | .11 |  |  |  |  |
| Plain | 29 | 5.2 | .6 | 4.3 | 6.0 | -.33 |  |  |  |  |
|  |  |  |  |  |  |  |  |  |  |  |
| PT * Views |  |  |  |  |  |  | 4.84 | .032^*^ | .077 |  |
| NoPT, Plain | 14 | 5.1 | .6 | 4.3 | 6.0 | -08 |  |  |  |  |
| NoPT, Rosie | 19 | 5.2 | .5 | 4.4 | 6.0 | -.65 |  |  |  |  |
| PT, Plain | 15 | 5.4 | .6 | 4.6 | 6.5 | 1.0 |  |  |  |  |
| PT, Rosie | 14 | 4.8 | .6 | 3.6 | 5.9 | -.32 |  |  |  |  |
| Plain: No v PT |  |  |  |  |  |  | 1.33 | .254 | .022 | -.17, .64 |
| Rosie: No v PT |  |  |  |  |  |  | 3.93 | .052^*^ | .064 | -.00, .77 |
| NoPT: Plain v Rosie |  |  |  |  |  |  | .35 | .556 | .006 | -.50, .27 |
| PT: Plain v Rosie |  |  |  |  |  |  | 6.10 | .017^*^ | .095 | .10, .91 |

*Note.* * = *p* ≤.05. The test was a repeated measures ANOVA two-tailed test. 95% Confidence Intervals are reported.

**Table 9**

*Descriptive and Inferential Statistics for Perspective Taking and Perceived Views on Subliminal Prime for Feminine Traits in Experiment 2*

| Comparison | *N* | *M* | *SD* | Min | Max | Skew | *F* | *p* | *η^2^* | *CI*s |
| --- | --- | --- | --- | --- | --- | --- | --- | --- | --- | --- |
| SubPrime |  |  |  |  |  |  | 1.77 | .156 | .039 |  |
| Self Fem + | 48 | 2.8 | .09 | 2.5 | 2.9 | -.28 |  |  |  |  |
| Self Fem - | 48 | 2.7 | 1.0 | 2.6 | 2.9 | .23 |  |  |  |  |
| NoSelf Fem + | 48 | 2.8 | 1.1 | 2.5 | 3.1 | -.05 |  |  |  |  |
| NoSelf Fem - | 48 | 2.8 | 1.0 | 2.6 | 3.1 | .82 |  |  |  |  |
|  |  |  |  |  |  |  |  |  |  |  |
| Prime * PT |  |  |  |  |  |  | .17 | .916 | .004 |  |
|  |  |  |  |  |  |  |  |  |  |  |
| Prime * Views |  |  |  |  |  |  | .45 | .717 | .010 |  |
|  |  |  |  |  |  |  |  |  |  |  |
| Prime*Views * PT |  |  |  |  |  |  | .52 | .607 | .014 |  |
| Self Fem +  NoPT, Plain | 11 | 2.7 | .07 | 2.6 | 2.8 | -.03 |  |  |  |  |
| Self Fem + | 13 | 2.8 | .09 | 2.6 | 2.9 | -.55 |  |  |  |  |
| NoPT, Rosie |  |  |  |  |  |  |  |  |  |  |
| Self Fem +   PT, Plain | 13 | 2.8 | .08 | 2.7 | 2.9 | .49 |  |  |  |  |
| Self Fem +  PT, Rosie | 11 | 2.7 | 1.0 | 2.5 | 2.9 | -.75 |  |  |  |  |
| Self Fem –  NoPT, Plain | 11 | 2.7 | .09 | 2.6 | 2.8 | .09 |  |  |  |  |
| Self Fem –  No PT, Rosie | 13 | 2.8 | 1.0 | 2.6 | 2.9 | -.08 |  |  |  |  |
| Self Fem –  PT, Plain | 13 | 2.7 | .10 | 2.6 | 2.9 | .57 |  |  |  |  |
| Self Fem –  PT, Rosie | 11 | 2.7 | 1.0 | 2.6 | 2.9 | .25 |  |  |  |  |
| No Self Fem +  NoPT, Plain | 11 | 2.8 | .11 | 2.5 | 2.9 | -.57 |  |  |  |  |
| No Self Fem +  NoPT, Rosie | 13 | 2.8 | .10 | 2.6 | 2.9 | -.32 |  |  |  |  |
| No Self Fem +  PT, Plain | 13 | 2.7 | .09 | 2.6 | 2.9 | .38 |  |  |  |  |
| No Self Fem +  PT, Rosie | 11 | 2.8 | .16 | 2.5 | 3.1 | .14 |  |  |  |  |
| No Self Fem –  NoPT, Plain | 11 | 2.8 | .08 | 2.7 | 2.9 | 1.0 |  |  |  |  |
| No Self Fem -   NoPT, Rosie | 13 | 2.8 | .11 | 2.6 | 3.0 | .03 |  |  |  |  |
| No Self Fem –  PT, Plain | 13 | 2.7 | .09 | 2.6 | 3.0 | .84 |  |  |  |  |
| No Self Fem -  PT, Rosie | 11 | 2.8 | .13 | 2.6 | 3.1 | 1.3 |  |  |  |  |
|  |  |  |  |  |  |  |  |  |  |  |

*Note.* * = *p* ≤.05. The test was a repeated measures ANOVA two-tailed test. 95% Confidence Intervals are reported.

**Table 10**

*Descriptive and Inferential Statistics for Perspective Taking and Perceived Views on Subliminal Prime for Masculine Traits in Experiment 2*

| Comparison | *N* | *M* | *SD* | Min | Max | Skew | *F* | *p* | *η^2^* |  |
| --- | --- | --- | --- | --- | --- | --- | --- | --- | --- | --- |
| SubPrime |  |  |  |  |  |  | 1.45 | .232 | .032 |  |
| Self Mas + | 48 | 2.8 | .10 | 2.5 | 3.0 | .20 |  |  |  |  |
| Self Mas - | 48 | 2.7 | .10 | 2.5 | 3.0 | .21 |  |  |  |  |
| NoSelf Mas + | 48 | 2.8 | .09 | 2.5 | 3.0 | .16 |  |  |  |  |
| NoSelf Mas - | 48 | 2.7 | .10 | 2.4 | 2.9 | -.47 |  |  |  |  |
|  |  |  |  |  |  |  |  |  |  |  |
| Prime * PT |  |  |  |  |  |  | 1.10 | .353 | .024 |  |
|  |  |  |  |  |  |  |  |  |  |  |
| Prime * Views |  |  |  |  |  |  | 1.21 | .309 | .027 |  |
|  |  |  |  |  |  |  |  |  |  |  |
| Prime * Views * PT |  |  |  |  |  |  | 1.38 | .253 | .030 |  |
| Self Mas +   NoPT, Plain | 11 | 2.8 | .10 | 2.6 | 2.9 | .16 |  |  |  |  |
| Self Mas + | 13 | 2.8 | .10 | 2.7 | 3.0 | .87 |  |  |  |  |
| NoPT, Rosie |  |  |  |  |  |  |  |  |  |  |
| Self Mas +  PT, Plain | 13 | 2.7 | .09 | 2.6 | 2.9 | .22 |  |  |  |  |
| Self Mas +  PT, Rosie | 11 | 2.8 | .14 | 2.5 | 3.0 | -.29 |  |  |  |  |
| Self Mas -   NoPT, Plain | 11 | 2.8 | .12 | 2.6 | 2.9 | .27 |  |  |  |  |
| Self Mas –  No PT, Rosie | 13 | 2.8 | .07 | 2.6 | 2.9 | .06 |  |  |  |  |
| Self Mas –  PT, Plain | 13 | 2.8 | .07 | 2.7 | 2.9 | .40 |  |  |  |  |
| Self Mas –  PT, Rosie | 11 | 2.7 | .15 | 2.5 | 3.0 | .29 |  |  |  |  |
| No Self Mas +   NoPT, Mas | 11 | 2.7 | .06 | 2.6 | 2.9 | .27 |  |  |  |  |
| No Self Mas +  NoPT, Rosie | 13 | 2.8 | .10 | 2.6 | 2.9 | .19 |  |  |  |  |
| No Self Mas +  PT, Plain | 13 | 2.8 | .09 | 2.7 | 2.9 | .59 |  |  |  |  |
| No Self Mas +  PT, Rosie | 11 | 2.7 | .12 | 2.5 | 3.0 | .15 |  |  |  |  |
| No Self Mas –  NoPT, Plain | 11 | 2.7 | .09 | 2.6 | 2.9 | .77 |  |  |  |  |
| No Self Mas -  NoPT, Rosie | 13 | 2.8 | .10 | 2.6 | 2.9 | -.63 |  |  |  |  |
| No Self Mas –  PT, Plain | 13 | 2.7 | .09 | 2.6 | 2.9 | .14 |  |  |  |  |
| No Self Mas -   PT, Rosie | 11 | 2.7 | .12 | 2.4 | 2.9 | -.98 |  |  |  |  |
|  |  |  |  |  |  |  |  |  |  |  |

*Note.* * = *p* ≤.05. The test was a repeated measures ANOVA two-tailed test. 95% Confidence Intervals are reported.

**Table 11**

*Descriptive and Inferential Statistics for Perspective Taking on Perspective Taking, Affiliation, and Self-Presentation in Experiment 2*

| DV  Comparison | | *N* | | *M* | | *SD* | *F* | *p* | *η^2^* | *CI*s |
| --- | --- | --- | --- | --- | --- | --- | --- | --- | --- | --- |
| **PT Measure** | |  | |  | |  |  |  |  |  |
| PT | |  | |  | |  | .13 | .716 | .002 | -.49, .70 |
| PT | | 29 | | 3.4 | | 1.2 |  |  |  |  |
| Control | | 33 | | 3.5 | | 1.1 |  |  |  |  |
|  | |  | |  | |  |  |  |  |  |
| Views | |  | |  | |  | .70 | .406 | .012 | -.35, .84 |
| Rosie | | 33 | | 3.3 | | 1.1 |  |  |  |  |
| Plain | | 29 | | 3.5 | | 1.2 |  |  |  |  |
|  | |  | |  | |  |  |  |  |  |
| PT*Views | |  | |  | |  | .20 | .653 | .004 |  |
| PT/Rosie | | 14 | | 3.2 | | 1.0 |  |  |  |  |
| PT/Plain | | 15 | | 3.5 | | 1.3 |  |  |  |  |
| NoPT/Rosie | | 19 | | 3.4 | | 1.1 |  |  |  |  |
| NoPT/Plain | | 14 | | 3.5 | | 1.2 |  |  |  |  |
|  | |  | |  | |  |  |  |  |  |
| **AM Measure** | |  | |  | |  |  |  |  |  |
| PT | |  | |  | |  | .00 | .980 | .000 | -.46, .47 |
| PT | | 29 | | 4.7 | | .89 |  |  |  |  |
| Control | | 33 | | 4.8 | | .89 |  |  |  |  |
|  | |  | |  | |  |  |  |  |  |
| Views | |  | |  | |  | .08 | .778 | .001 | -.40, .53 |
| Rosie | | 33 | | 4.7 | | .85 |  |  |  |  |
| Plain | | 29 | | 4.8 | | .94 |  |  |  |  |
|  | |  | |  | |  |  |  |  |  |
| PT*Views | |  | |  | |  | .80 | .376 | .014 |  |
| PT/Rosie | | 14 | | 4.6 | | .69 |  |  |  |  |
| PT/Plain | | 15 | | 4.9 | | 1.1 |  |  |  |  |
| NoPT/Rosie | | 19 | | 4.8 | | .95 |  |  |  |  |
| NoPT/Plain | | 14 | | 4.7 | | .84 |  |  |  |  |
|  | |  | |  | |  |  |  |  |  |
| **Self-Present** | |  | |  | |  |  |  |  |  |
| PT | |  | |  | |  | 1.18 | .282 | .020 | -.27, .92 |
| PT | 29 | | | | 2.6 | 1.2 |  |  |  |  |
| Control | 33 | | | | 3.0 | 1.1 |  |  |  |  |
|  |  | | | |  |  |  |  |  |  |
| Views |  | | | |  |  | .02 | .899 | .000 | -56, .64 |
| Rosie | 33 | | | | 2.8 | 1.1 |  |  |  |  |
| Plain | 29 | | | | 2.8 | 1.2 |  |  |  |  |
|  |  | | | |  |  |  |  |  |  |
| PT*Views | |  | | |  |  | .36 | .551 | .001 |  |
| PT/Rosie | | | 14 | | 2.8 | 1.2 |  |  |  |  |
| PT/Plain | | | 15 | | 2.5 | 1.2 |  |  |  |  |
| NoPT/Rosie | | | 17 | | 2.9 | 1.1 |  |  |  |  |
| NoPT/Plain | | | 17 | | 3.0 | 1.2 |  |  |  |  |
|  | |  | |  | |  |  |  |  |  |

*Note.* * = *p* ≤.05. The analyses were two-way ANOVAs with a two-tailed test. 95% Confidence Intervals are reported.

**Table 12**

*Descriptive and Inferential Statistics for Perspective Taking and Perceived Views on Explicit Egalitarian Attitudes in Experiment 3*

| Comparison | *N* | *M* | *SD* | Min | Max | Skew | *F* | *p* | *η^2^* | *CI*s |
| --- | --- | --- | --- | --- | --- | --- | --- | --- | --- | --- |
| PT |  |  |  |  |  |  | .62 | .431 | .006 | -.21, .48 |
| PT | 54 | 5.4 | 1.0 | 3.1 | 6.9 | -.36 |  |  |  |  |
| No PT | 55 | 5.3 | .9 | 3.7 | 7.0 | .13 |  |  |  |  |
|  |  |  |  |  |  |  |  |  |  |  |
| Views |  |  |  |  |  |  | .16 | .693 | .001 | -.27, .41 |
| Eracism | 54 | 5.4 | 1.0 | 3.7 | 7.0 | .10 |  |  |  |  |
| Plain | 55 | 5.3 | .8 | 3.1 | 7.0 | -.19 |  |  |  |  |
|  |  |  |  |  |  |  |  |  |  |  |
| PT * Views |  |  |  |  |  |  | 4.02 | .048^*^ | .037 |  |
| NoPT, Plain | 25 | 5.4 | .8 | 3.9 | 6.9 | -01 |  |  |  |  |
| NoPT, Erace | 30 | 5.1 | .9 | 3.1 | 6.9 | -.39 |  |  |  |  |
| PT, Plain | 30 | 5.2 | .9 | 3.7 | 7.0 | .26 |  |  |  |  |
| PT, Erace | 24 | 5.6 | 1.0 | 4.0 | 7.0 | -.09 |  |  |  |  |
| Plain: No v PT |  |  |  |  |  |  | .75 | .390 | .007 | -.27, .69 |
| Erace: No v PT |  |  |  |  |  |  | 3.86 | .052^*^ | .035 | -.00, .97 |
| NoPT: Plain v Erace |  |  |  |  |  |  | 2.85 | .094 | .026 | -.07, .90 |
| PT: Plain v Erace |  |  |  |  |  |  | 1.30 | .255 | .012 | -.20, .76 |
|  |  |  |  |  |  |  |  |  |  |  |

*Note.* * = *p* ≤.05. The analysis was a two-way ANOVA with a two-tailed test. 95% Confidence Intervals are reported.

**Table 13**

*Descriptive and Inferential Statistics for Perspective Taking and Perceived Views on Implicit Egalitarian Attitudes in Experiment 3*

| Comparison | *N* | *M* | *SD* | Min | Max | Skew | *F* | *p* | *η^2^* | *CI*s |
| --- | --- | --- | --- | --- | --- | --- | --- | --- | --- | --- |
| PT |  |  |  |  |  |  | .08 | .784 | .001 | -.13, .18 |
| PT | 24 | -.6 | .4 | -1.1 | .48 | .62 |  |  |  |  |
| No PT | 30 | -.5 | .5 | -1.6 | .24 | .38 |  |  |  |  |
|  |  |  |  |  |  |  |  |  |  |  |
| Views |  |  |  |  |  |  | .14 | .705 | .001 | -.12, .18 |
| Eracism | 24 | -.6 | .4 | -1.6 | .30 | -.43 |  |  |  |  |
| Plain | 30 | -.5 | .4 | -1.3 | .48 | .70 |  |  |  |  |
|  |  |  |  |  |  |  |  |  |  |  |
| PT * Views |  |  |  |  |  |  | .50 | .480 | .005 |  |
| NoPT, Plain | 24 | -.5 | .4 | -1.1 | .30 | .33 |  |  |  |  |
| NoPT, Erace | 30 | -.5 | .5 | -1.1 | .48 | .72 |  |  |  |  |
| PT, Plain | 30 | -.5 | .4 | -1.6 | .09 | -.98 |  |  |  |  |
| PT, Erace | 24 | -.6 | .4 | -1.3 | .24 | .55 |  |  |  |  |
| Plain: No v PT |  |  |  |  |  |  | 1.0 | .785 | .001 | -.25, .18 |
| Erace: No v PT |  |  |  |  |  |  | .48 | .490 | .005 | -.14, 29 |
| NoPT:Plain v Erace |  |  |  |  |  |  | .06 | .815 | .001 | -.19, .24 |
| PT: Plain v Erace |  |  |  |  |  |  | .59 | .446 | .006 | -.13, .30 |
|  |  |  |  |  |  |  |  |  |  |  |

*Note.* * = *p* ≤.05. The test was a two-way ANOVA with a two-tailed test. 95% Confidence Intervals are reported.

**Table 14**

*Descriptive and Inferential Statistics for Perspective Taking and Perceived Views on Perspective Taking, Affiliation, and Self-Presentation in Experiment 3*

| DV  Comparison | *N* | | *M* | *SD* | *F* | *p* | *η^2^* | *CI*s |
| --- | --- | --- | --- | --- | --- | --- | --- | --- |
| **PT Measure** |  | |  |  |  |  |  |  |
| PT |  | |  |  | 1.23 | .271 | .012 | -.22, .77 |
| PT | 54 | | 4.0 | 1.3 |  |  |  |  |
| Control | 55 | | 3.7 | 1.3 |  |  |  |  |
|  |  | |  |  |  |  |  |  |
| Views |  | |  |  | .05 | .833 | .000 | -.44, .54 |
| Erace | 54 | | 3.8 | 1.3 |  |  |  |  |
| Plain | 55 | | 3.9 | 1.3 |  |  |  |  |
|  |  | |  |  |  |  |  |  |
| PT*Views |  | |  |  | .01 | .928 | .000 |  |
| PT/Erace | 24 | | 4.0 | 1.3 |  |  |  |  |
| PT/Plain | 30 | | 4.0 | 1.2 |  |  |  |  |
| NoPT/Erace | 30 | | 3.7 | 1.3 |  |  |  |  |
| NoPT/Plain | 25 | | 3.7 | 1.3 |  |  |  |  |
|  |  | |  |  |  |  |  |  |
| **AM Measure** |  | |  |  |  |  |  |  |
| PT |  | |  |  | 5.24 | .024* | .048 | .05, .69 |
| PT | 54 | | 5.6 | .8 |  |  |  |  |
| Control | 55 | | 5.2 | .9 |  |  |  |  |
|  |  | |  |  |  |  |  |  |
| Views |  | |  |  | .40 | .531 | .004 | -.22, .42 |
| Erace | 54 | | 5.4 | .8 |  |  |  |  |
| Plain | 55 | | 5.4 | .9 |  |  |  |  |
|  |  | |  |  |  |  |  |  |
| PT*Views |  | |  |  | .68 | .412 | .006 |  |
| PT/Erace | 24 | | 5.7 | .8 |  |  |  |  |
| PT/Plain | 30 | | 5.5 | .8 |  |  |  |  |
| NoPT/Erace | 30 | | 5.2 | .9 |  |  |  |  |
| NoPT/Plain | 25 | | 5.3 | .9 |  |  |  |  |
|  |  | |  |  |  |  |  |  |
| **Self-Present** |  | |  |  |  |  |  |  |
| PT |  | |  |  | .23 | .634 | .002 | -.39, .64 |
| PT | 54 | | 2.8 | 1.4 |  |  |  |  |
| Control | 55 | | 3.0 | 1.3 |  |  |  |  |
|  |  | |  |  |  |  |  |  |
| Views |  | |  |  | .03 | .875 | .000 | -.48, .56 |
| Erace | 54 | | 2.9 | 1.4 |  |  |  |  |
| Plain | 55 | | 2.9 | 1.3 |  |  |  |  |
|  |  | |  |  |  |  |  |  |
| PT*Views | |  |  |  | .09 | .763 | .001 |  |
| PT/Erace | | 24 | 2.9 | 1.4 |  |  |  |  |
| PT/Plain | | 30 | 2.8 | 1.4 |  |  |  |  |
| NoPT/Erace | | 30 | 2.9 | 1.4 |  |  |  |  |
| NoPT/Plain | | 25 | 3.0 | 1.3 |  |  |  |  |
|  |  | |  |  |  |  |  |  |

*Note.* * = *p* ≤.05. The analysis was a two-way ANOVA with a two-tailed test. 95% Confidence Intervals are reported.

**Table 15**

*Descriptive and Inferential Statistics for Perspective Taking and Stated Expectations on Explicit Egalitarian Attitudes in Experiment 4*

| Comparison | *N* | *M* | *SD* | Min | Max | Skew | *F* | *p* | *η^2^* | *CI*s |
| --- | --- | --- | --- | --- | --- | --- | --- | --- | --- | --- |
| PT |  |  |  |  |  |  | .36 | .552 | .004 | -.22, .40 |
| PT | 43 | 4.9 | .8 | 3.3 | 7.0 | .19 |  |  |  |  |
| No PT | 40 | 4.8 | .7 | 3.7 | 6.4 | .40 |  |  |  |  |
|  |  |  |  |  |  |  |  |  |  |  |
| Expectations |  |  |  |  |  |  | .26 | .615 | .003 | -.23, .23 |
| Egalitarian | 41 | 4.8 | .7 | 3.3 | 6.4 | .07 |  |  |  |  |
| Prejudice | 42 | 4.9 | .8 | 3.7 | 7.0 | .45 |  |  |  |  |
|  |  |  |  |  |  |  |  |  |  |  |
| PT * Views |  |  |  |  |  |  | 5.17 | .026^*^ | .061 |  |
| NoPT, Prejudice | 21 | 4.7 | .6 | 3.7 | 5.9 | .41 |  |  |  |  |
| NoPT, Egalitarian | 19 | 5.0 | .8 | 3.7 | 6.4 | .19 |  |  |  |  |
| PT, Prejudice | 21 | 5.1 | .8 | 3.7 | 7.0 | .14 |  |  |  |  |
| PT, Egalitarian | 22 | 4.7 | .6 | 3.3 | 6.0 | -.52 |  |  |  |  |
| Prejudice: No v PT |  |  |  |  |  |  | 4.18 | .044^*^ | .050 | .01, .88 |
| Egalitarian: No v PT |  |  |  |  |  |  | 1.39 | .243 | .017 | -.18, .70 |
| NoPT: Prej v Egal |  |  |  |  |  |  | 4.01 | .049^*^ | .048 | .00, .86 |
| PT: Prej v Egal |  |  |  |  |  |  | 1.51 | .223 | .019 | -.17, .72 |
|  |  |  |  |  |  |  |  |  |  |  |

*Note.* * = *p* ≤.05. The analysis was a two-way ANOVA with a two-tailed test. 95% Confidence Intervals are reported.

**Table 16**

*Descriptive and Inferential Statistics for Perspective Taking and Stated Expectations on Implicit Egalitarian Attitudes in Experiment 4*

| Comparison | *N* | *M* | *SD* | Min | Max | Skew | *F* | *p* | *η^2^* | *CI*s |
| --- | --- | --- | --- | --- | --- | --- | --- | --- | --- | --- |
| PT |  |  |  |  |  |  | .149 | .701 | .002 | -.12, .17 |
| PT | 43 | .48 | .30 | -.1 | 1.2 | -.09 |  |  |  |  |
| No PT | 40 | .48 | .31 | -.6 | 1.0 | -.77 |  |  |  |  |
|  |  |  |  |  |  |  |  |  |  |  |
| Expectations |  |  |  |  |  |  | .347 | .557 | .004 | -.10, .19 |
| Egalitarian | 42 | .45 | .35 | -.1 | 1.0 | -.55 |  |  |  |  |
| Prejudice | 41 | .49 | .30 | -.6 | 1.2 | -.37 |  |  |  |  |
|  |  |  |  |  |  |  |  |  |  |  |
| PT * Expectations |  |  |  |  |  |  | .235 | .629 | .003 |  |
| NoPT, Prejudice | 21 | .41 | .41 | -.6 | .9 | -.85 |  |  |  |  |
| NoPT, Egalitarian | 19 | .49 | .28 | .0 | 1.0 | -.04 |  |  |  |  |
| PT, Prejudice | 30 | .48 | .30 | -.0 | 1.2 | .53 |  |  |  |  |
| PT, Egalitarian | 22 | .48 | .31 | -.1 | .9 | -.59 |  |  |  |  |
|  |  |  |  |  |  |  |  |  |  |  |
|  |  |  |  |  |  |  |  |  |  |  |

*Note.* * = *p* ≤.05. The analysis was a two-way ANOVA with a two-tailed test. 95% Confidence Intervals are reported.

**Table 17**

*Descriptive and Inferential Statistics for Perspective Taking and Stated Expectations on Perspective Taking, Affiliation, and Self-Presentation in Experiment 4*

| DV  Comparison | *N* | | *M* | *SD* | *F* | *p* | *η^2^* | | *CI*s | | |  |  |
| --- | --- | --- | --- | --- | --- | --- | --- | --- | --- | --- | --- | --- | --- |
| **PT Measure** |  | |  |  |  |  |  | |  | | |  |  |
| PT |  | |  |  | .60 | .443 | .007 | | -.33, .74 | | |  |  |
| PT | 43 | | 3.9 | 1.1 |  |  |  | |  | | |  |  |
| Control | 40 | | 4.1 | 1.3 |  |  |  | |  | | |  |  |
|  |  | |  |  |  |  |  | |  | | |  |  |
| Expectation |  | |  |  | .20 | .654 | .003 | | -.41, .65 | | |  |  |
| Egalitarian | 41 | | 4.1 | 1.3 |  |  |  | |  | | |  |  |
| Prejudice | 42 | | 4.0 | 1.2 |  |  |  | |  | | |  |  |
|  |  | |  |  |  |  |  | |  | | |  |  |
| PT*Expect |  | |  |  | .02 | .892 | .000 | |  | | |  |  |
| PT/Egalitarian | 22 | | 4.0 | 1.1 |  |  |  | |  | | |  |  |
| PT/Prejudice | 21 | | 3.8 | 1.1 |  |  |  | |  | | |  |  |
| NoPT/Egalitarian | 19 | | 4.2 | 1.5 |  |  |  | |  | | |  |  |
| NoPT/Prejudice | 21 | | 4.1 | 1.2 |  |  |  | |  | | |  |  |
|  |  | |  |  |  |  |  | |  | | |  |  |
| **AM Measure** |  | |  |  |  |  |  | |  | | |  |  |
| PT |  | |  |  | .08 | .778 | .001 | | -.35, .46 | | |  |  |
| PT | 43 | | 5.6 | 1.0 |  |  |  | |  | | |  |  |
| Control | 40 | | 5.5 | .80 |  |  |  | |  | | |  |  |
|  |  | |  |  |  |  |  | |  | | |  |  |
| Expectation |  | |  |  | .60 | .440 | .008 | | -.25, .56 | | |  |  |
| Egalitarian | 41 | | 5.6 | .99 |  |  |  | |  | | |  |  |
| Prejudice | 42 | | 5.4 | .84 |  |  |  | |  | | |  |  |
|  |  | |  |  |  |  |  | |  | | |  |  |
| PT*Expect |  | |  |  | .01 | .915 | .000 | |  | | |  |  |
| PT/Egalitarian | 22 | | 5.6 | 1.1 |  |  |  | |  | | |  |  |
| PT/Prejudice | 21 | | 5.4 | .93 |  |  |  | |  | | |  |  |
| NoPT/Egalitarian | 19 | | 5.6 | .85 |  |  |  | |  | | |  |  |
| NoPT/Prejudice | 21 | | 5.6 | .76 |  |  |  | |  | | |  |  |
|  |  | |  |  |  |  |  | |  | | |  |  |
| **Self-Present** |  | |  |  |  |  |  | |  | | |  |  |
| PT |  | |  |  | .39 | .536 | .005 | | -.49, .94 | | |  |  |
| PT | 43 | | 3,0 | 1.6 |  |  |  | |  | | |  |  |
| Control | 40 | | 3.2 | 1.7 |  |  |  | |  | | |  |  |
|  |  | |  |  |  |  |  | |  | | |  |  |
| Expectation |  | |  |  | .08 | .776 | .001 | | -.62, .82 | | |  |  |
| Egalitarian | 41 | | 3.1 | 1.8 |  |  |  | |  | | |  |  |
| Prejudice | 42 | | 3.0 | 1.5 |  |  |  | |  | | |  |  |
|  |  | |  |  |  |  |  | |  | | |  |  |
| PT*Expect | |  |  |  | .06 | .814 | .001 | |  | | |  |  |
| PT/Egalitarian | | 22 | 3.1 | 1.7 |  |  |  | |  | | |  |  |
| PT/Prejudice | | 21 | 2.9 | 1.4 |  |  |  | |  | | |  |  |
| NoPT/Egalitarian | | 19 | 3.2 | 1.9 |  |  |  | |  | | |  |  |
| NoPT/Prejudice | | 21 | 3.2 | 1.6 |  |  |  | |  | | |  |  |
|  |  | |  |  |  |  |  |  | |  |  | |  |

*Note.* * = *p* ≤.05. The analyses were two-way ANOVAs with a two-tailed test. 95% Confidence Intervals are reported.

**Table 18**

*Descriptive and Inferential Statistics for Perspective Taking and Ostensible IAT Results on Explicit Egalitarian Attitudes in Experiment 5*

| Comparison | *N* | *M* | *SD* | Min | Max | Skew | *F* | *p* | *η^2^* | *CI*s |
| --- | --- | --- | --- | --- | --- | --- | --- | --- | --- | --- |
| PT |  |  |  |  |  |  | 2.03 | .159 | .029 | -.12, .73 |
| PT | 37 | 4.6 | .74 | 3.2 | 6.2 | -.03 |  |  |  |  |
| No PT | 38 | 4.4 | 1.1 | 1.7 | 7.0 | -.62 |  |  |  |  |
|  |  |  |  |  |  |  |  |  |  |  |
| IAT Fake Result |  |  |  |  |  |  | .64 | .428 | .009 | -.27, .62 |
| Egalitarian | 37 | 4.6 | .74 | 3.1 | 7.0 | .42 |  |  |  |  |
| Prejudice | 38 | 4.4 | .78 | 1.7 | 6.2 | -.90 |  |  |  |  |
|  |  |  |  |  |  |  |  |  |  |  |
| PT * Views |  |  |  |  |  |  | 4.42 | .039^*^ | .060 |  |
| NoPT, Prejudice | 17 | 4.0 | 1.3 | 1.7 | 6.1 | -.69 |  |  |  |  |
| NoPT, Egalitarian | 21 | 4.7 | .88 | 3.1 | 7.0 | .56 |  |  |  |  |
| PT, Prejudice | 21 | 4.7 | .80 | 3.4 | 6.2 | -.03 |  |  |  |  |
| PT, Egalitarian | 16 | 4.5 | .64 | 3.2 | 5.5 | -.42 |  |  |  |  |
| Prejudice: No v PT |  |  |  |  |  |  | 6.23 | .015^*^ | .083 | .15, 1.4 |
| Egalitarian: No v PT |  |  |  |  |  |  | .23 | .630 | .003 | -.46, .75 |
| NoPT: Prej v Egal |  |  |  |  |  |  | 4.38 | .040^*^ | .060 | 03, 1.2 |
| PT: Prej v Egal |  |  |  |  |  |  | .74 | .391 | .011 | -.36, .91 |
|  |  |  |  |  |  |  |  |  |  |  |
| IV Order Covariate |  |  |  |  |  |  | .42 | .518 | .006 |  |
| DV Order Covariate |  |  |  |  |  |  | 3.59 | .062 | .049 |  |
|  |  |  |  |  |  |  |  |  |  |  |

*Note.* * = *p* ≤.05. The analysis was a two-way ANOVA with a two-tailed test. 95% Confidence Intervals are reported.

**Table 19**

*Descriptive and Inferential Statistics for Perspective Taking and Ostensible IAT Results on Implicit Egalitarian Attitudes in Experiment 5*

| Comparison | *N* | *M* | *SD* | Min | Max | Skew | *F* | *p* | *η^2^* | *CI*s |
| --- | --- | --- | --- | --- | --- | --- | --- | --- | --- | --- |
| PT |  |  |  |  |  |  | .02 | .904 | .000 | -15, 18 |
| PT | 37 | 4.8 | 36.5 | 139 | 8 | -.32 |  |  |  |  |
| No PT | 38 | 8.4 | 36.0 | 84 | 8 | 1.3 |  |  |  |  |
|  |  |  |  |  |  |  |  |  |  |  |
| IAT Fake Result |  |  |  |  |  |  | 1.11 | .296 | .016 | -8, 27 |
| Egalitarian | 37 | 12.6 | 36.8 | -53 | 139 | 1.2 |  |  |  |  |
| Prejudice | 38 | .7 | 34.8 | -102 | 84 | -.29 |  |  |  |  |
|  |  |  |  |  |  |  |  |  |  |  |
| PT * Views |  |  |  |  |  |  | .80 | .375 | .011 |  |
| NoPT, Prejudice | 17 | -1.4 | 24.4 | -54 | 46 | .08 |  |  |  |  |
| NoPT, Egalitarian | 21 | 16.3 | 42.9 | -53 | 139 | 1.1 |  |  |  |  |
| PT, Prejudice | 21 | 2.5 | 41.9 | -102 | 84 | -.43 |  |  |  |  |
| PT, Egalitarian | 16 | 7.8 | 27.4 | -44 | 63 | .72 |  |  |  |  |
| Prejudice: No v PT |  |  |  |  |  |  | .30 | .586 | .004 | -17, 31 |
| Egalitarian: No v PT |  |  |  |  |  |  | .51 | .476 | .007 | -15, 33 |
| NoPT:Prej v Egal |  |  |  |  |  |  | 2.0 | .161 | .028 | -6, 41 |
| PT: Prej v Egal |  |  |  |  |  |  | .02 | .893 | .000 | -23, 27 |
|  |  |  |  |  |  |  |  |  |  |  |
| IV Order Covariate |  |  |  |  |  |  | .10 | .752 | .001 |  |
| DV Order Covariate |  |  |  |  |  |  | 1.78 | .187 | .025 |  |
|  |  |  |  |  |  |  |  |  |  |  |

*Note.* * = *p* ≤.05. The analysis was a two-way ANOVA with a two-tailed test. 95% Confidence Intervals are reported.

**Table 20**

*Descriptive and Inferential Statistics for Perspective Taking and Ostensible IAT Results on Perspective Taking, Affiliation, and Self-Presentation in Experiment 5*

| DV  Comparison | *N* | | *M* | *SD* | *F* | *p* | *η^2^* | *CI*s |
| --- | --- | --- | --- | --- | --- | --- | --- | --- |
| **PT Measure** |  | |  |  |  |  |  |  |
| PT |  | |  |  | .23 | .632 | .003 | -.48, .78 |
| PT | 37 | | 3.4 | 1.2 |  |  |  |  |
| Control | 36 | | 3.6 | 1.4 |  |  |  |  |
|  |  | |  |  |  |  |  |  |
| IAT Fake Result |  | |  |  | .20 | .656 | .003 | -.51, .81 |
| Egalitarian | 36 | | 3.6 | 1.4 |  |  |  |  |
| Prejudice | 37 | | 3.5 | 1.2 |  |  |  |  |
|  |  | |  |  |  |  |  |  |
| PT*Expect |  | |  |  | .84 | .364 | .012 |  |
| PT/Egalitarian | 16 | | 3.3 | 1.3 |  |  |  |  |
| PT/Prejudice | 21 | | 3.5 | 1.1 |  |  |  |  |
| NoPT/Egalitarian | 20 | | 3.8 | 1.5 |  |  |  |  |
| NoPT/Plain | 16 | | 3.4 | 1.4 |  |  |  |  |
|  |  | |  |  |  |  |  |  |
| IV Order Covariate |  | |  |  | .13 | .720 | .002 |  |
| DV Order Covariate |  | |  |  | .29 | .591 | .004 |  |
|  |  | |  |  |  |  |  |  |
| **AM Measure** |  | |  |  |  |  |  |  |
| PT |  | |  |  | .16 | .690 | .002 | -.37, .55 |
| PT | 37 | | 4.6 | 1.0 |  |  |  |  |
| Control | 36 | | 4.7 | 1.0 |  |  |  |  |
|  |  | |  |  |  |  |  |  |
| IAT Fake Result |  | |  |  | .29 | .595 | .004 | -.35, .61 |
| Egalitarian | 36 | | 4.7 | 1.0 |  |  |  |  |
| Prejudice | 37 | | 4.6 | .94 |  |  |  |  |
|  |  | |  |  |  |  |  |  |
| PT*Expect |  | |  |  | 5.31 | .024^*^ | .073 |  |
| PT/Egalitarian | 16 | | 4.3 | 1.1 |  |  |  |  |
| PT/Prejudice | 21 | | 4.8 | .84 |  |  |  |  |
| NoPT/Egalitarian | 20 | | 5.0 | .93 |  |  |  |  |
| NoPT/Plain | 16 | | 4.3 | 1.0 |  |  |  |  |
| Prejudice: No v PT |  | |  |  | 1.80 | .184 | .026 | -.21, 1.1 |
| Egalitarian: No v PT |  | |  |  | 3.67 | .060 | .052 | -.03, 1.3 |
| NoPT:Prej v Egal |  | |  |  | 4.02 | .049^*^ | .057 | .00, 1.3 |
| PT: Prej v Egal |  | |  |  | 1.42 | .238 | .021 | -.27, 1.1 |
|  |  | |  |  |  |  |  |  |
| IV Order Covariate |  | |  |  | .02 | .885 | .000 |  |
| DV Order Covariate |  | |  |  | .36 | .553 | .005 |  |
|  |  | |  |  |  |  |  |  |
| **Self-Present** |  | |  |  |  |  |  |  |
| PT |  | |  |  | .13 | .723 | .002 | -.51, .73 |
| PT | 37 | | 2.7 | 1.3 |  |  |  |  |
| Control | 36 | | 2.6 | 1.3 |  |  |  |  |
|  |  | |  |  |  |  |  |  |
| IAT Fake Result |  | |  |  | .12 | .734 | .002 | -.54, .76 |
| Egalitarian | 36 | | 2.7 | 1.3 |  |  |  |  |
| Prejudice | 37 | | 2.7 | 1.3 |  |  |  |  |
|  |  | |  |  |  |  |  |  |
| PT*Expect | |  |  |  | 1.83 | .181 | .027 |  |
| PT/Egalitarian | | 16 | 2.5 | 1.4 |  |  |  |  |
| PT/Prejudice | | 21 | 2.9 | 1.2 |  |  |  |  |
| NoPT/Egalitarian | | 20 | 2.8 | 1.3 |  |  |  |  |
| NoPT/Plain | | 16 | 2.3 | 1.4 |  |  |  |  |
|  | |  |  |  |  |  |  |  |
| IV Order Covariate |  | |  |  | .07 | .796 | .001 |  |
| DV Order Covariate |  | |  |  | .87 | .355 | .013 |  |
|  |  | |  |  |  |  |  |  |

*Note.* * = *p* ≤.05. The analyses were two-way ANOVAs with a two-tailed test. 95% Confidence Intervals are reported.
